# *Clostridium difficile* Lipoprotein GerS Is Required for Cortex Modification and Thus Spore Germination

**DOI:** 10.1128/mSphere.00205-18

**Published:** 2018-06-27

**Authors:** Oscar R. Diaz, Cameron V. Sayer, David L. Popham, Aimee Shen

**Affiliations:** a Department of Molecular Biology and Microbiology, Tufts University School of Medicine, Boston, Massachusetts, USA; b NIH Postbaccalaureate Research Education Program (PREP), Tufts University School of Medicine, Boston, Massachusetts, USA; c Department of Biological Sciences, Virginia Tech, Blacksburg, Virginia, USA; University of Iowa

**Keywords:** *Clostridium difficile*, CwlD, GerS, PdaA, cortex modification, germination, lipoprotein

## Abstract

Clostridium difficile, also known as Clostridioides difficile, is a Gram-positive, spore-forming bacterium that is a leading cause of antibiotic-associated diarrhea. C. difficile infections begin when its metabolically dormant spores germinate to form toxin-producing vegetative cells. Successful spore germination depends on the degradation of the cortex, a thick layer of modified peptidoglycan that maintains dormancy. Cortex degradation is mediated by the SleC cortex lytic enzyme, which is thought to recognize the cortex-specific modification muramic-δ-lactam. C. difficile cortex degradation also depends on the *Peptostreptococcaceae*-specific lipoprotein GerS for unknown reasons. In this study, we tested whether GerS regulates production of muramic-δ-lactam and thus controls the ability of SleC to recognize its cortex substrate. By comparing the muropeptide profiles of Δ*gerS* spores to those of spores lacking either CwlD or PdaA, both of which mediate cortex modification in Bacillus subtilis, we determined that C. difficile GerS, CwlD, and PdaA are all required to generate muramic-δ-lactam. Both GerS and CwlD were needed to cleave the peptide side chains from N-acetylmuramic acid, suggesting that these two factors act in concert. Consistent with this hypothesis, biochemical analyses revealed that GerS and CwlD directly interact and that CwlD modulates GerS incorporation into mature spores. Since Δ*gerS*, Δ*cwlD*, and Δ*pdaA* spores exhibited equivalent germination defects, our results indicate that C. difficile spore germination depends on cortex-specific modifications, reveal GerS as a novel regulator of these processes, and highlight additional differences in the regulation of spore germination in C. difficile relative to B. subtilis and other spore-forming organisms.

**IMPORTANCE** The Gram-positive, spore-forming bacterium Clostridium difficile is a leading cause of antibiotic-associated diarrhea. Because C. difficile is an obligate anaerobe, its aerotolerant spores are essential for transmitting disease, and their germination into toxin-producing cells is necessary for causing disease. Spore germination requires the removal of the cortex, a thick layer of modified peptidoglycan that maintains spore dormancy. Cortex degradation is mediated by the SleC hydrolase, which is thought to recognize cortex-specific modifications. Cortex degradation also requires the GerS lipoprotein for unknown reasons. In our study, we tested whether GerS is required to generate cortex-specific modifications by comparing the cortex composition of Δ*gerS* spores to the cortex composition of spores lacking two putative cortex-modifying enzymes, CwlD and PdaA. These analyses revealed that GerS, CwlD, and PdaA are all required to generate cortex-specific modifications. Since loss of these modifications in Δ*gerS*, Δ*cwlD*, and Δ*pdaA* mutants resulted in spore germination and heat resistance defects, the SleC cortex lytic enzyme depends on cortex-specific modifications to efficiently degrade this protective layer. Our results further indicate that GerS and CwlD are mutually required for removing peptide chains from spore peptidoglycan and revealed a novel interaction between these proteins. Thus, our findings provide new mechanistic insight into C. difficile spore germination.

## Abstract (precis)

The Gram-positive, spore-forming bacterium Clostridium difficile is a leading cause of antibiotic-associated diarrhea. Because C. difficile is an obligate anaerobe, its aerotolerant spores are essential for transmitting disease, and their germination into toxin-producing cells is necessary for causing disease. Spore germination requires the removal of the cortex, a thick layer of modified peptidoglycan that maintains spore dormancy. Cortex degradation is mediated by the SleC hydrolase, which is thought to recognize cortex-specific modifications. Cortex degradation also requires the GerS lipoprotein for unknown reasons. In our study, we tested whether GerS is required to generate cortex-specific modifications by comparing the cortex composition of Δ*gerS* spores to the cortex composition of spores lacking two putative cortex-modifying enzymes, CwlD and PdaA. These analyses revealed that GerS, CwlD, and PdaA are all required to generate cortex-specific modifications. Since loss of these modifications in Δ*gerS*, Δ*cwlD*, and Δ*pdaA* mutants resulted in spore germination and heat resistance defects, the SleC cortex lytic enzyme depends on cortex-specific modifications to efficiently degrade this protective layer. Our results further indicate that GerS and CwlD are mutually required for removing peptide chains from spore peptidoglycan and revealed a novel interaction between these proteins. Thus, our findings provide new mechanistic insight into C. difficile spore germination.

## INTRODUCTION

Clostridium difficile, which was reclassified as Clostridioides difficile (1), is a Gram-positive, spore-forming obligate anaerobe that is the leading microbial cause of health care-associated infections in the United States (2, 3). The CDC reports that ~500,000 cases of C. difficile infections occur each year in the United States, contributing to ~30,000 deaths (4). Prolonged broad-spectrum antibiotic treatment can increase susceptibility to infection due to disruption of the colonization resistance provided by our gut microbiota (5, 6). Part of this resistance is mediated by gut bacteria that transform primary bile acids into secondary bile acids, since these metabolic transformations inhibit C. difficile vegetative cell growth and may decrease the efficiency of spore germination (6–9).

C. difficile produces two large clostridial toxins, TcdA and TcdB, that disrupt the actin cytoskeleton and epithelial tight junctions and can induce cell death (10). The toxins’ cytopathic and cytotoxic effects cause disease pathologies ranging from mild diarrhea to severe pseudomembranous colitis. However, even though C. difficile pathogenesis depends on toxin production, its ability to transmit disease relies on its ability to form and germinate spores, because C. difficile is an obligate anaerobe (11). Furthermore, the formation of resistant, aerotolerant, metabolically dormant spores by C. difficile allows it to bypass the effects of antibiotic exposure in the gut and to persist in the environment for long periods of time.

The multilayered structure of bacterial spores provides protection against environmental insults and helps them maintain their metabolically dormant state. The outer proteinaceous layer, known as the coat, acts as a molecular sieve to help spores resist enzymatic and oxidative insults (12). Beneath the coat is a thick layer of modified peptidoglycan known as the cortex, which exists between two membranes known as the outer and inner forespore membranes (13, 14). The cortex consists of a thick outer layer of modified peptidoglycan on top of the thin innermost germ cell wall layer; this thin layer becomes the outgrowing cell wall of germinating spores (15). The cortex surrounds the spore core, which contains its genetic material and consists of partially dehydrated cytosol. The low water content of the core combined with the protection of spore DNA by the activity of small, acid-soluble proteins (SASPs) helps to maintain spores in a metabolically dormant state (16). The cortex is critical for maintaining this partially dehydrated state because it acts like a vice to prevent expansion of the core through hydration. As a result, spore germination depends upon the thick cortex layer being enzymatically removed by dedicated cortex lytic enzymes.

Previous work has established the cortex lytic enzyme, SleC, as the major enzyme responsible for degrading the cortex layer during C. difficile spore germination (17–21). SleC is conserved in many clostridial organisms (22), and its activity is controlled by regulated proteolysis, which removes the inhibitory N-terminal propeptide of SleC in C. perfringens (23, 24) and C. difficile (18). The Csp family proteases are essential for this proteolytic activation event in both C. perfringens and C. difficile. However, there are major differences between C. perfringens and C. difficile in the signaling pathway that leads to SleC activation. In C. perfringens, any one of three Csp proteases, CspA, CspB, or CspC, can proteolytically activate SleC (24). In contrast, in C. difficile, only CspB is proteolytically active because both C. difficile CspA and CspC carry catalytic site mutations (18). Furthermore, unlike C. perfringens, the CspA pseudoprotease is produced as a C-terminal fusion to the CspB protease in C. difficile (18, 19, 25), and the CspC pseudoprotease regulates CspB protease activity by directly sensing bile acid germinants (26).

These differences appear to be conserved at the family level because the CspC pseudoprotease and CspA pseudoprotease domains are conserved in the members of the *Peptostreptococcaceae* family, whereas the members of the *Clostridiaceae* and *Lachnospiraceae* families encode catalytically competent CspC and CspA proteases (19). While it is unclear whether CspC pseudoproteases function as germinant receptors in other *Peptostreptococcaceae* family members, the catalytic mutations in CspC are strictly conserved (19). We recently identified an additional *Peptostreptococcaceae* family-specific protein required for C. difficile spore germination: the GerS lipoprotein (20). Using Targetron-based gene disruption of *gerS*, we showed that loss of C. difficile GerS leads to a >4-log reduction in spore germination in the JIR8094 strain background (27, 28) because the cortex is not degraded (20). Interestingly, although C. difficile *gerS* mutant spores fail to hydrolyze their cortex, the pro-SleC zymogen is still proteolytically processed in response to germinants (20).

These observations suggest that C. difficile GerS is required for spore germination because it (i) modulates SleC cortex lytic enzyme activity downstream of CspB-mediated proteolytic cleavage or (ii) is necessary to generate the cortex-specific peptidoglycan modifications that are presumably needed by SleC to recognize and degrade the cortex layer. Previous studies in Bacillus subtilis have shown that muramic-δ-lactam (MAL) residues distinguish spore cortex peptidoglycan from the germ cell wall (29, 30). Approximately half the N-acetylmuramic acid (NAM) residues in B. subtilis cortex peptidoglycan are converted to MAL residues, while ~25% carry tetrapeptide side chains and the remainder have single d-Ala side chains due to the action of the LytH hydrolase (31).

**FIG 1:**
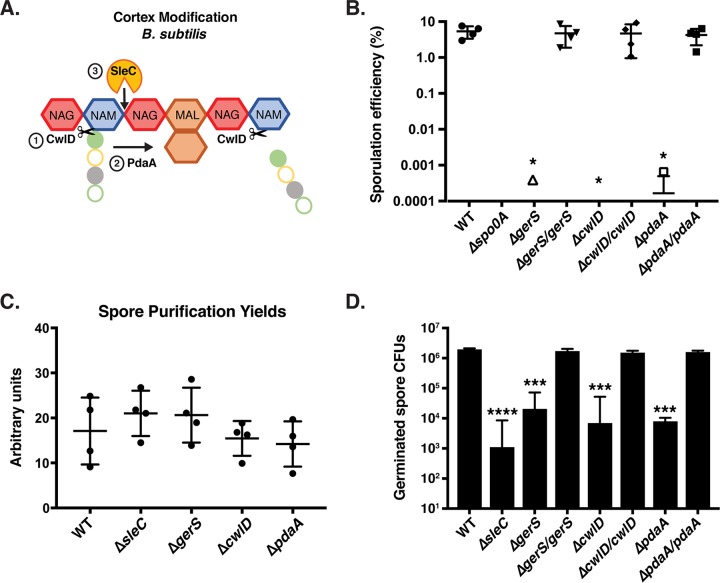
*gerS*, *cwlD*, and *pdaA* mutants exhibit germination defects but form wild-type levels of spores. (A) Schematic of cortex peptidoglycan modifications mediated by CwlD and PdaA in B. subtilis. Red hexagons represent N-acetylglucosamine (NAG); blue hexagons represent N-acetylmuramic acid (NAM); brown hexagons represent muramic-δ-lactam (MAL); filled green circles represent l-alanine; open yellow circles represent d-glutamic acid; filled gray circles represent m-2,6-diaminopimelic acid; open green circles represent d-alanine. The order of CwlD function relative to PdaA function on peptidoglycan is shown. (B) Apparent sporulation efficiencies of the wild-type (WT), Δ*gerS*, Δ*cwlD*, and Δ*pdaA* strains and their complements measured for four biological replicates by heat-treating sporulation cultures and determining CFU on media containing germinant cultures relative to untreated cultures. The Δ*spo0A* mutant served as a negative control because it cannot form spores (11). Averages of results from four biological replicates are shown along with the associated standard deviations. (C) Spore purification yields. Sporulating cells were harvested from 70:30 agar plates 48 h following plating. The optical density at 600 nm for all purified spore stocks resuspended in identical 600-µl volumes was determined divided by the total number of 70:30 plates used per strain. Averages and standard deviations of results from three independent experiments are shown. (D) Germination efficiency of Δ*gerS*, Δ*cwlD*, and Δ*pdaA* spores and their complements. Averages of results from three biological replicates performed using two independent spore preparations are shown along with the associated standard deviations. The Δ*sleC* mutant served as a negative control because it is defective in cortex hydrolysis (21). Statistical analyses were performed using one-way ANOVA and Tukey’s test. *, *P* < 0.05; **, *P* < 0.005; ***, *P* < 0.0005; ****, *P* < 0.0001; n.s., not statistically significant.

The MAL modification is specifically recognized by cortex lytic enzymes, ensuring that these lytic enzymes do not degrade the vegetative germ cell wall of spores (29, 32, 33). In B. subtilis, two enzymes act sequentially on spore peptidoglycan to generate MAL residues: CwlD, an N-acetylmuramoyl-l-alanine amidase, and PdaA, an N-acetylmuramic acid deacetylase (34, 35) (Fig. 1A). CwlD removes the tetrapeptide side chains attached to NAM residues, while PdaA deacetylates the NAM residues and performs lactam cyclization to produce MAL (34–37). Loss of either CwlD or PdaA in B. subtilis leads to severe defects in cortex degradation and thus to spore germination on media containing germinants but not germination events upstream of cortex degradation (34, 35, 37).

In this study, we sought to test whether GerS is necessary for generating the muramic-δ-lactam (MAL) cortex-specific modifications that are presumably recognized by SleC. In order to test this hypothesis, we compared the muropeptide profiles of spores lacking either of the putative cortex-modifying enzymes CwlD and PdaA to spores lacking GerS. Since C. difficile CwlD and PdaA share 57% and 54% sequence similarity to their B. subtilis homologs (see Fig. S1 in the supplemental material), respectively, they likely have functions similar to those of their B. subtilis counterparts. The germination phenotypes and peptidoglycan compositions of *gerS*, *cwlD*, and *pdaA* mutant spores were compared to provide the first insights (to our knowledge) into the mechanisms controlling cortex modification in the Clostridia and their impact on spore physiology.

10.1128/mSphere.00205-18.1 FIG S1  ClustalW alignment of CwlD (A) and PdaA (B). Conserved identical residues are shown in white and blocked in black, while conserved similar residues are shown in gray. The putative signal peptide cleavage site and several predicted important residues (Asp 73, His 124, His 128, Arg 166, and His 222) for B. subtilis PdaA have been boxed in red based on the crystal structure of B. subtilis PdaA. Accession numbers for CwlD homologs are as follows: Clostridioides difficile (YP_001087931.1), Clostridioides mangenotii (WP_027701768.1), Paraclostridium bifermentans (WP_021432562.1), Paeniclostridium sordellii (WP_055333993.1), Clostridium perfringens (WP_003470288.1), Bacillus subtilis (WP_003244439.1), Bacillus anthracis (WP_071728789.1), and Paenibacillus tuaregi (WP_082927025.1). Accession numbers for PdaA homologs are as follows: Clostridioides difficile (YP_001087931.1), Clostridioides mangenotii (WP_027701768.1), Paraclostridium bifermentans (WP_021432562.1), Paeniclostridium sordellii (WP_055333993.1), Clostridium perfringens (WP_003470288.1), Bacillus subtilis (WP_003244439.1), Bacillus anthracis (WP_071728789.1), Paenibacillus tuaregi (WP_082927025.1), and Paenibacillus sp. DMB20 (WP_046677656.1). Download FIG S1, TIF file, 2.9 MB. Copyright © 2018 Diaz et al. 2018 Diaz et al. This content is distributed under the terms of the Creative Commons Attribution 4.0 International license.

## RESULTS

### Characterization of Δ*gerS*, Δ*cwlD*, and Δ*pdaA* mutants.

To construct mutations in the *gerS*, *cwlD*, and *pdaA* genes, we used *pyrE*-based allele-coupled exchange (ACE) to make clean deletions (see Fig. S2 in the supplemental material) (38). Unlike the Targetron-based gene disruption method that we previously used to construct a *gerS*::*ermB* mutant in the JIR8094 strain background, the clean deletions prevented polar effects on downstream gene expression. Although mutation of the alanine racemase (*alr2*) gene downstream of *gerS* does not strongly alter germination (20, 39), constructing a *gerS* in-frame deletion avoids effects on downstream gene expression. Another advantage of the *pyrE-*based ACE system is that it allows single-copy, chromosomal complementation of the *gerS* (C. difficile 630_34640 [CD630_34640]), *cwlD* (CD630_01060), and *pdaA* (CD630_14300) mutations and avoids introducing experimental artifacts associated with the overexpression of plasmid-based complementation constructs (19).

10.1128/mSphere.00205-18.2 FIG S2  Generation of Δ*gerS*, Δ*cwlD*, and Δ*pdaA* mutants by *pyrE*-based ACE (38). A schematic of the allelic exchange used to construct gene deletions is shown. Strain construction was confirmed by colony PCR of Δ*gerS*, Δ*cwlD*, and Δ*pdaA* strains using primers that flank the gene of interest and C. difficile 630 genomic DNA as a control. Download FIG S2, TIF file, 2.2 MB. Copyright © 2018 Diaz et al. 2018 Diaz et al. This content is distributed under the terms of the Creative Commons Attribution 4.0 International license.

To characterize these mutant strains, we determined their apparent sporulation efficiency levels by measuring CFU levels on media containing germinants following heat treatment of sporulating cultures. Specifically, Δ*gerS*, Δ*cwlD*, and Δ*pdaA* strains were induced to sporulate, and the resulting samples were either heated to kill any vegetative cells or left untreated and then plated on media containing taurocholate (40). The Δ*gerS*, Δ*cwlD* and Δ*pdaA* mutants all exhibited an ~5-log decrease in apparent sporulation efficiency relative to the wild type (*P* < 0.02, Fig. 1B). In fact, no CFU were detected in three of the four biological replicates performed, while CFU were detected in the three mutant strains at a frequency of ~0.0005% in the fourth biological replicate, which is close to the limit of detection of this assay. The apparent sporulation efficiency of the 630Δ*erm* Δ*gerS* strain was roughly equivalent to that previously reported for the JIR8094 *gerS*::*ermB* Targetron mutant (20), while the apparent sporulation efficiency of Δ*cwlD* was similar to that of the previously constructed 630Δ*erm* Δ*cwlD* strain (41). Importantly, complementing the Δ*gerS*, Δ*cwlD*, and Δ*pdaA* strains with wild-type alleles of *gerS*, *cwlD* and *pdaA*, respectively, from the *pyrE* locus restored the apparent sporulation efficiency of the mutants to wild-type levels (Fig. 1B).

Since the apparent sporulation efficiency measured using the heat-treatment assay does not distinguish between defects in heat resistance, spore formation, germination, and/or outgrowth, we tested whether Δ*gerS*, Δ*cwlD*, and Δ*pdaA* spores would be purified as efficiently as wild-type spores. The spore purification yield for each strain was measured by inducing sporulation, purifying spores using density gradient centrifugation, resuspending spores in equivalent volumes, and determining their yields by measuring optical density at 600 nm (OD_600_). No significant differences in purified spore yields were observed for the Δ*gerS*, Δ*cwlD*, and Δ*pdaA* strains relative to the wild-type strain or the germination-defective Δ*sleC* mutant (42) (Fig. 1C).

When Δ*gerS*, Δ*cwlD*, and Δ*pdaA* spores were plated on media containing germinant, an ~2-to-3-log decrease in spore germination efficiency was observed in the mutants relative to the wild-type strain (*P* value < 0.0001, Fig. 1D). Δ*pdaA* spores exhibited a 5-fold-greater germination defect than the Δ*gerS* and Δ*cwlD* spores, but this difference was not statistically significant. The Δ*sleC* mutant, which lacks the primary cortex lytic SleC enzyme, exhibited a 3-log decrease in germination efficiency (*P* < 0.0001, Fig. 1D) as previously reported (25, 42). Notably, Δ*gerS*, Δ*cwlD*, and Δ*pdaA* spores all exhibited delayed germination phenotypes, producing colonies from germinating spores more slowly than wild-type colonies and with kinetics similar to Δ*sleC* spore kinetics (25). In addition, as previously observed with C. difficile Δ*sleC* and Δ*cspBAC* mutants (25, 42) and B. subtilis germination receptor mutants (43), the germination efficiencies for Δ*gerS*, Δ*cwlD*, and Δ*pdaA* spores differed between spore preparations for unknown reasons, so all analyses were performed on two independent biological replicate preparations of purified spores. While the 2-log germination defect of 630Δ*erm* Δ*gerS* spores was considerably less severe than the 4-log germination defect reported for our JIR8094 *gerS*::*ermB* mutant (20), this discrepancy is consistent with the ~2-to-3-log-less-severe germination defects observed for *sleC* and *cspBAC* Targetron mutant spores in the 630Δ*erm* strain relative to the JIR8094 strain background (25). Although the reason for this difference is unclear, these results indicate that GerS, CwlD, and PdaA are critical for spore germination. Notably, the germination defects of Δ*gerS*, Δ*cwlD*, and Δ*pdaA* spores (~2 to 3 logs; Fig. 1D) were markedly less severe than their ~5-log defect in apparent sporulation efficiency (Fig. 1B), suggesting that GerS, CwlD, and PdaA contribute to spore heat resistance.

To test this possibility, we exposed purified Δ*gerS*, Δ*cwlD*, and Δ*pdaA* spores to 60°C heat treatment, similarly to the sporulating cell analyses, and measured the capacity of heat-treated spores to germinate and form colonies on media containing germinant. While the germinated CFU levels for wild-type and Δ*sleC* spores were unchanged by the 60°C treatment, Δ*gerS*, Δ*cwlD*, and Δ*pdaA* germinated CFU levels decreased by ~10-fold (*P* ≤ 0.05, Fig. S3A), which was similar to the approximately −5-fold decrease previously reported for the *gerS*::*ermB* mutant in JIR8094 (20). The apparent increase in the heat sensitivity of Δ*gerS*, Δ*cwlD*, and Δ*pdaA* mutant spores relative to wild-type spores was not due to decreased dipicolinic acid (DPA) levels (44), since measurement of spore DPA levels revealed that Δ*gerS* and Δ*cwlD* spores had slightly higher levels of DPA than wild-type spores (*P* < 0.05, Fig. S3B).

10.1128/mSphere.00205-18.3 FIG S3  Apparent heat sensitivity of Δ*gerS*, Δ*cwlD*, Δ*pdaA*, and Δ*sleC* spores. (A) CFU produced by germinated spores after 23 h on BHIS-TA. The spores were either left untreated or subjected to heat treatment for 15 min at 60°C. Statistical analysis included two-way ANOVA and Tukey’s test. (B) Measurement of total DPA levels in wild-type and Δ*gerS*, Δ*cwlD*, Δ*pdaA*, and Δ*sleC* mutant spores. The amount of DPA within these spores was measured using terbium fluorescence after the spores were treated at 37°C or 95°C for 1 h. Statistical analysis included one-way ANOVA and Tukey’s test. Averages of results from three independent experiments performed using two independent spores are shown, and the error bars indicate the standard deviation for each measurement. *, *P* ≤ 0.05; **, *P* < 0.005. Download FIG S3, TIF file, 1.2 MB. Copyright © 2018 Diaz et al. 2018 Diaz et al. This content is distributed under the terms of the Creative Commons Attribution 4.0 International license.

### Abnormal forespore morphology in *cwlD* and *pdaA* mutants.

**FIG 2:**
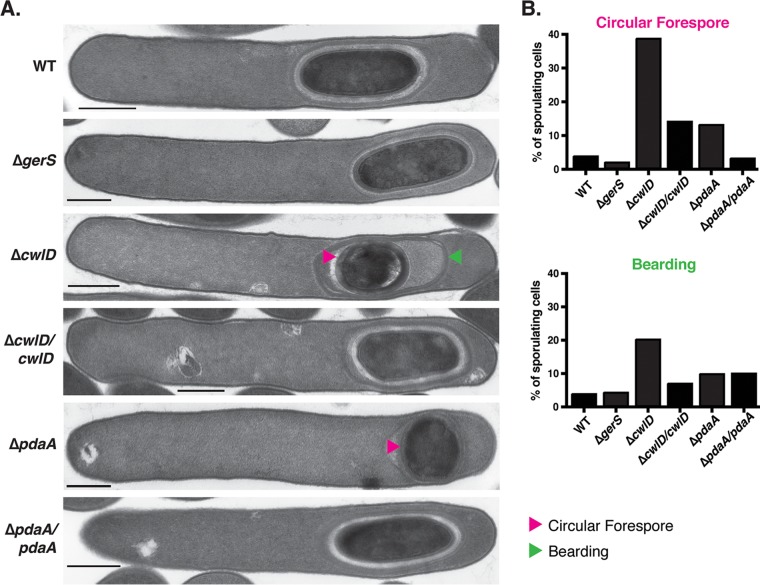
Abnormal forespore morphology in Δ*cwlD* and Δ*pdaA* sporulating cells. (A) TEM analyses of sporulating cells of the wild-type, Δ*gerS*, Δ*cwlD* and complement, and Δ*pdaA* and complement strains at 23 h. Pink arrowheads mark circular forespores, and the green arrowhead marks an instance of bearding. Bars, 500 nm (B) Percentages of sporulating cells that displayed circular forespore morphology or bearding are shown based on analyses of 50 cells, except for the Δ*cwlD cwlD* mutant, for which the data represent analysis of 25 cells.

Interestingly, while analyzing sporulation in these mutants by phase-contrast microscopy, we observed that the Δ*cwlD* and Δ*pdaA* mutants produced forespores with an abnormal, circular morphology (as opposed to an ovoid morphology) more frequently than the wild-type strain and the Δ*gerS* mutant (Fig. S4). To visualize these morphological defects with greater resolution, we analyzed sporulating cells of the mutants and their complements using transmission electron microscopy (TEM). A minimum of 50 sporulating cells of each mutant was examined 23 h after sporulation induction. Circular forespores were observed in 39% and 13% of Δ*cwlD* and Δ*pdaA* sporulating cells, respectively (Fig. 2A), which represent 10-fold and 3-fold increases over wild-type cells consistent with our phase-contrast microscopy analyses (Fig. S4). The apparent increase in the frequency of circular forespores indicated by TEM relative to phase-contrast microscopy was likely a function of the higher degree of confidence in scoring cells as circular forespores. Importantly, complementation of the Δ*cwlD* and Δ*pdaA* mutants reduced the frequency of circular forespores by ~3-fold. Taking the results together, the microscopy analyses indicate that the putative cortex-modifying enzymes, CwlD and PdaA, help generate and/or maintain wild-type spore morphology.

10.1128/mSphere.00205-18.4 FIG S4  (A) Phase-contrast microscopy of the indicated C. difficile strains cultured in sporulation media for 22 h. The Δ*spo0A* mutant cannot undergo sporulation. Pink arrows highlight abnormally shaped circular, phase-bright forespores. Yellow arrows indicate the ovoid, phase-bright forespores. The apparent level of sporulation efficiency is shown below relative to the wild-type level. Averages and standard deviations of results from four independent experiments are shown. (B) Frequency of abnormal circular phase-bright (fore)spore formation relative to all sporulating cells. Quantification of phase-contrast microscopy images was performed for a minimum of 450 sporulating cells per strain across three biological replicates. Statistical analysis included one-way ANOVA and Tukey’s test, and the standard deviations are shown for each measurement. ***, *P* < 0.0005. Download FIG S4, TIF file, 1.3 MB. Copyright © 2018 Diaz et al. 2018 Diaz et al. This content is distributed under the terms of the Creative Commons Attribution 4.0 International license.

Interestingly, ~50% of the circular forespores observed in the Δ*cwlD* mutant also exhibited coat tethering defects where the coat appeared to slough off the forespore (also known as “bearding” [45]). Coat detachment was observed in only 1 of 33 Δ*cwlD* forespores with ovoid morphology relative to 10 of 21 circular Δ*cwlD* forespores. This bearding phenomenon was elevated in Δ*cwlD* (20%) and Δ*pdaA* (10%) sporulating cells but not Δ*gerS* sporulating cells (5%) relative to the wild type (4%) (Fig. 2B). While it is unclear why coat localization defects were observed in the Δ*cwlD* and Δ*pdaA* strains, the observation that bearding correlates with circular forespore formation in the Δ*cwlD* mutant could imply that geometric cues play a role in tethering the coat to the forespore. In analyses of purified spores by TEM, the Δ*gerS*, Δ*cwlD*, and Δ*pdaA* spores appeared qualitatively similar to wild-type spores. The spore cortex of the mutant strains, however, appeared darker (more electron dense) than the wild type (Fig. S5). This darkened cortex phenotype for the Δ*cwlD* spores relative to the wild type has previously been observed by TEM in B. subtilis (33), so the more electron-dense cortex of the C. difficile mutant spores may indicate that GerS, CwlD, and PdaA are required to modify the cortex.

10.1128/mSphere.00205-18.5 FIG S5  Spore morphology and germination efficiency of purified Δ*gerS*, Δ*cwlD*, and Δ*pdaA* spores. (A) Phase-contrast microscopy of purified spores for the indicated C. difficile strains. The Δ*sleC* strain is defective in cortex degradation (21) and thus in spore germination, although a low level was observed (termed “spontaneous germination” [25]). Germination efficiency (GE) levels relative to the wild-type levels are shown. Pink arrows highlight circular, phase-bright spores. Statistical significance was determined using a one-way ANOVA with Tukey’s test. Averages of results from three biological replicates performed using two independent spore preparations are shown along with the associated standard deviations. (n.s., not statistically significant.) (B) TEM of wild-type, Δ*gerS*, Δ*cwlD*, and Δ*pdaA* spores. Representative images of cross sections of spores from the indicated strains are shown. Scale bars represent 100 nm. Download FIG S5, TIF file, 1.6 MB. Copyright © 2018 Diaz et al. 2018 Diaz et al. This content is distributed under the terms of the Creative Commons Attribution 4.0 International license.

### GerS, CwlD, and PdaA are necessary for cortex degradation.

**FIG 3:**
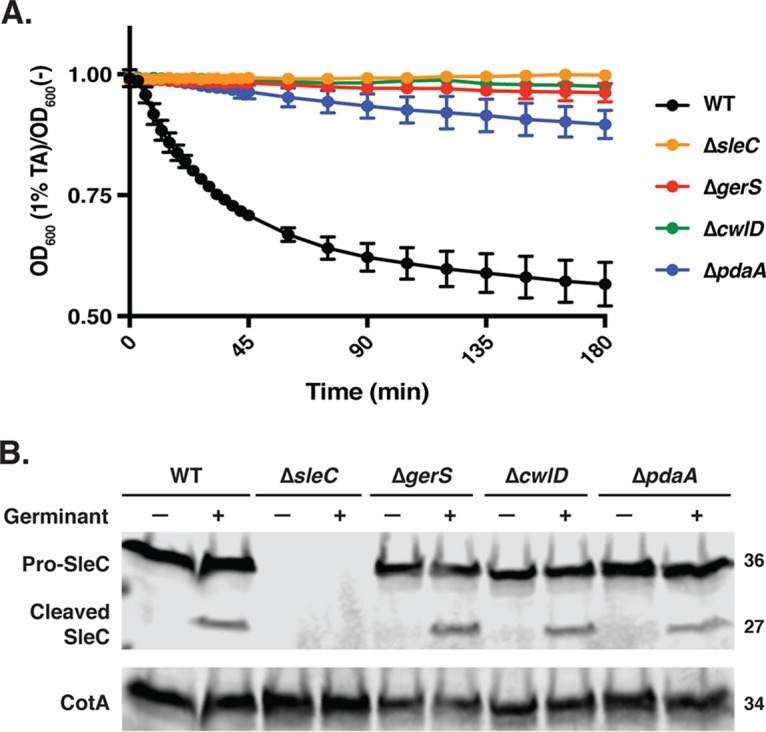
Δ*gerS*, Δ*cwlD*, and Δ*pdaA* spores are defective in cortex degradation but not SleC cleavage. (A) Purified spores were suspended in BHIS, taurocholate was added at a final concentration of 1%, and the optical density (OD_600_) of each sample was measured out to 3 h. Statistical analysis was completed using repeated-measures ANOVA and Tukey’s test. Averages of results from three independent experiments performed using two independent spore preparations are shown, and the error bars indicate the standard deviation for each time point measured. (B) Western blot analysis of purified spores of one representative replicate following taurocholate treatment.

Given that B. subtilis *cwlD* and *pdaA* mutants are defective in spore germination because their cortex lytic enzymes, CwlJ and SleB, cannot recognize their substrate MAL (33), we tested whether cortex degradation was impaired in C. difficile mutants lacking *gerS*, *cwlD*, and *pdaA* using an optical-density-based assay. Cortex degradation can be indirectly assessed by measuring the change in optical density at 600 nm (OD_600_) of spores as they germinate, hydrate, and thus decrease in optical density. Previous work has shown that cortex degradation constitutes the initial 20% drop in OD_600_ observed in wild-type germinating spores and that the subsequent 20% drop can be attributed to core rehydration (44). The optical density of wild-type spores rapidly decreased in the initial 45 min after taurocholate addition and then remained largely stable after 3 h, while the optical density of Δ*sleC* spores remained unchanged over this time period because SleC is necessary for cortex degradation (21). The optical density of Δ*cwlD* spores did not significantly decrease over 3 h, while Δ*gerS* and Δ*pdaA* spores exhibited slight but significant drops at 2.5 and 1 h, respectively, following taurocholate treatment (Fig. 3A). These observations suggest that Δ*gerS*, Δ*cwlD*, and Δ*pdaA* mutant strains are defective in cortex degradation to differing degrees. While the inability to detect a delayed germination phenotype for Δ*cwlD* spores may reflect the low sensitivity of the OD_600_ germination drop assay, the delayed germination phenotype of Δ*gerS* and Δ*pdaA* spores in the optical density assay is consistent with the qualitatively delayed germination observed in plate-based germination assays (data not shown).

To assess whether the cortex degradation defects in Δ*gerS*, Δ*cwlD*, and Δ*pdaA* spores were due to an inability to process the pro-SleC zymogen into its catalytically active form, we analyzed SleC cleavage following treatment with taurocholate. As expected, the predominant form of SleC observed in untreated samples was the pro-SleC zymogen for all strains. Exposure of wild-type, Δ*gerS*, Δ*cwlD*, and Δ*pdaA* spores to taurocholate resulted in similar levels of SleC processing (Fig. 3B). These results confirm our previous analyses of the *gerS*::*ermB* strain in the JIR8094 strain background (20), but they contrast with recent studies of a 630Δ*erm ΔgerS* mutant (46) where SleC cleavage was shown to decrease in the absence of GerS. However, the latter studies used different experimental conditions to assess SleC cleavage, and no complementation analyses of the Δ*gerS* strain were performed. Regardless, our observations strongly suggest that Δ*gerS*, Δ*cwlD*, and Δ*pdaA* spores are defective in spore germination because they inefficiently hydrolyze the cortex despite proteolytically activating SleC.

### GerS, CwlD, and PdaA are required to generate the cortex-specific modification muramic-δ-lactam.

**FIG 4:**
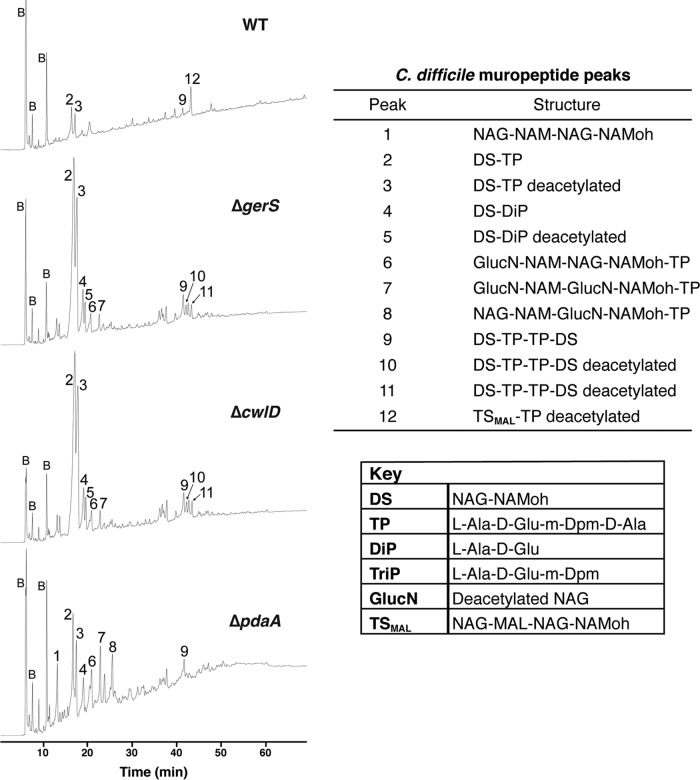
GerS and CwlD are necessary for removal of peptidoglycan (PG) peptide side chains; PdaA is necessary for deacetylation. C. difficile spore cortex peptidoglycan was purified and digested using mutanolysin. The resulting muropeptides were reduced, separated by reversed-phase HPLC, and identified by mass spectrometry. Peaks are numbered as indicated in Table S1; the peaks labeled "B" represent buffer components. "NAG" represents N-acetylglucosamine, "NAM" represents N-acetylmuramic acid, "MAL" represents muramic-δ-lactam, and "NAMoh" represents reduced N-acetylmuramic acid.

The results thus far were consistent with the hypothesis that Δ*gerS*, Δ*cwlD*, and Δ*pdaA* mutants cannot degrade the cortex because they fail to generate the cortex-specific MAL modifications required for SleC to recognize its cortex substrate. This hypothesis is based on the observation that B. subtilis cortex lytic enzymes require MAL residues for cortex degradation to proceed (33) and that B. subtilis CwlD and PdaA are sequentially required to generate MAL residues (34, 35). To directly test this hypothesis, we analyzed the muropeptide profiles of our mutant strains. Cortex peptidoglycan was isolated from wild-type, Δ*gerS*, Δ*cwlD*, and Δ*pdaA* spores and digested with muramidase, which cleaves between NAM and N-acetylglucosamine (NAG) residues but not between MAL and NAG residues (30, 47). The digestion products were separated using reverse-phase high-performance liquid chromatography (HPLC), and the identities of the muropeptide compounds within individual peaks were determined using mass spectrometry (MS) (Fig. 4; see also Fig. S6 and Table S1 in the supplemental material for details regarding peak assignments).

10.1128/mSphere.00205-18.6 FIG S6  Example MS analysis of muropeptide 12. Purified muropeptides were analyzed using MALDI-TOF-TOF mass spectrometry as described in Materials and Methods. (A) Primary MS spectra of muropeptide 12, revealing the major Na+ parent ion at *m*/*z* = 1,340.3; a doubly charged Na+H+ ion was observed at *m*/*z* = 739.2. (B) MS-MS spectrum of muropeptide 12 fragmented parent ion at *m*/*z* = 1,340.3. The identities of major fragment ions are shown in Table S1. Download FIG S6, PDF file, 0.1 MB. Copyright © 2018 Diaz et al. 2018 Diaz et al. This content is distributed under the terms of the Creative Commons Attribution 4.0 International license.

10.1128/mSphere.00205-18.8 TABLE S1  MS-MS analyses of muropeptide structures. Download TABLE S1, PDF file, 0.1 MB. Copyright © 2018 Diaz et al. 2018 Diaz et al. This content is distributed under the terms of the Creative Commons Attribution 4.0 International license.

Several peaks were identified in the peptidoglycan isolated from all four mutant spores: these peaks corresponded to the disaccharide (DS, NAG-NAM) carrying the l-Ala-d-Glu-m-Dpm-d-Ala tetrapeptide (TP, with "m-Dpm" referring to meso-2,6-diaminopimelic acid) (peak 2) and to two DS-TP (disaccharide tetrapeptides) cross-linked together (peak 9). A large fraction of the DS-TP was deacetylated on the NAG residue (GlucN, peak 3), consistent with the observation that large portions of NAG (N-acetylglucosamine) residues are deacetylated in vegetative C. difficile peptidoglycan (48).

Notably, a single peak (peak 12) was unique to the peptidoglycan isolated from wild-type spores. Peak 12 corresponds to a tetrasaccharide containing MAL (TS_MAL_), the distinguishing feature of cortex peptidoglycan (33). Approximately one-third of peptidoglycan fragments from the wild type contained this cortex-specific modification (Fig. 4). Since this peak was absent from Δ*gerS*, Δ*cwlD*, and Δ*pdaA* spores, these results indicate that GerS, CwlD, and PdaA are all required for MAL synthesis and thus presumably for SleC to recognize its cortex substrate.

Remarkably, the muropeptide profile of Δ*cwlD* spores was identical to that of Δ*gerS* spores in peak presence and intensity. Peaks 2 and 3 increased markedly in Δ*gerS* and Δ*cwlD* spores relative to wild-type and Δ*pdaA* spores. These peaks contain disaccharide tetrapeptides (DS-TP) with and without acetylation, respectively, the major muropeptide expected if the putative amidase activity of C. difficile CwlD prevents removal of peptide side chains as previously observed for B. subtilis Δ*cwlD* spores (33, 49). The relative increases in peaks 2 and 3 resulted from the lack of MAL in Δ*gerS* and Δ*cwlD* peptidoglycan, which allows mutanolysin to produce more DS species. Increases in peaks 4 and 5 may result from the dramatically increased abundance of peaks 2 and 3, providing more substrate for an unidentified endopeptidase that cleaves the peptide side chains. Regardless, since the muropeptide profiles of Δ*gerS* and Δ*cwlD* spores were essentially indistinguishable, these results strongly suggest that GerS is required for CwlD amidase activity to generate the substrate required for SleC recognition. Alternatively, GerS may directly modify the cortex in a CwlD-dependent manner.

Two muropeptides were unique to the Δ*pdaA* strain. Peak 1 consists of a tetrasaccharide (TS) that lacks any peptide side chains and contains no MAL. This species corresponds to the predicted product of CwlD-mediated removal of the tetrapeptide and is consistent with analyses of B. subtilis CwlD produced in Escherichia coli (35). Peak 8 contained the second muropeptide exclusively produced in the Δ*pdaA* mutant, a tetrasaccharide tetrapeptide variant that differs from peaks 6 and 7 by the position and extent of NAG deacetylation. Notably, Δ*pdaA* spores did not accumulate the large amounts of the DS-TP variants (peaks 2 and 3) observed in Δ*gerS* and Δ*cwlD* spores, presumably because CwlD (and/or GerS) removes some of the peptide side chains. Taking the data together, the muropeptide profile analyses indicate that GerS and CwlD activities are coupled and mediate tetrapeptide removal, whereas PdaA likely acts downstream of GerS and CwlD in modifying the peptide side chain to generate muramic-δ-lactam residues.

### CwlD is critical for GerS signal peptide processing and incorporation into mature spores.

**FIG 5:**
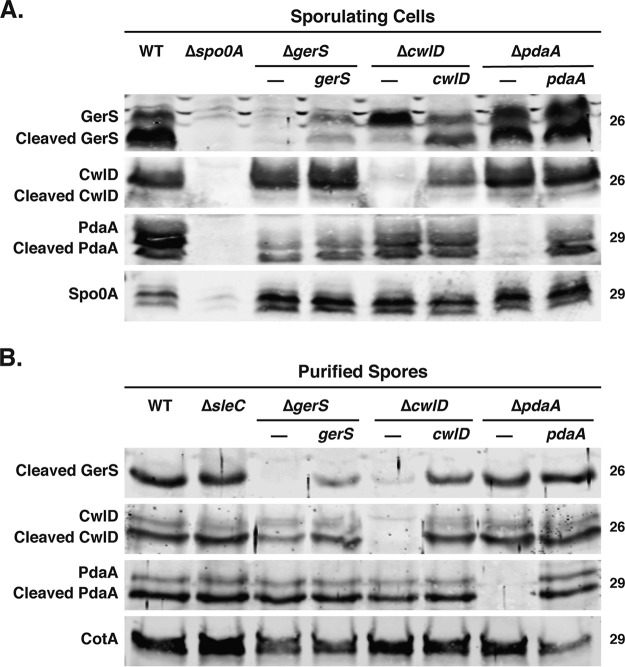
CwlD alters GerS processing and incorporation into spores. (A) Western blot analysis of sporulating cells from the wild-type (WT), Δ*gerS*, Δ*cwlD*, and Δ*pdaA* strains and their complements. The Δ*spo0A* mutant served as a negative control because it cannot form spores (11), and Spo0A was used as a loading control (61). (B) Western blot analysis of the spores that the strains used in the experiments described in the panel A legend. The Δ*sleC* mutant served as a negative control for spore germination (25, 42). Anti-CotA was used as a loading control.

Since the *gerS* and *cwlD* mutants produced identical spore muropeptide profiles and shared similar levels of spore heat sensitivity and germination defects, the putative amidase activity of CwlD appears to depend on GerS. To gain insight into the relationship between GerS and CwlD, we tested whether GerS might affect CwlD levels in sporulating cells and/or purified spores and vice versa. Our previous plasmid-based complementation analyses of a *gerS* Targetron mutant revealed that GerS follows a typical Gram-positive lipoprotein biogenesis pathway (50) in which GerS is translocated across the outer forespore membrane by its N-terminal signal peptide, becomes lipidated on a conserved cysteine (Cys22), and then is processed to remove its signal peptide, leaving the protein tethered to the membrane by virtue of its N-terminal lipidation (20, 51). Accordingly, both full-length GerS and cleaved GerS were detectable in wild-type sporulating cells, while only cleaved GerS was detected in mature spores (20). Both full-length GerS and cleaved GerS were detected in sporulating Δ*cwlD* and Δ*pdaA* cells, but the proportion of full-length GerS was dramatically higher in Δ*cwlD* sporulating cells than in Δ*pdaA* and wild-type cells (Fig. 5A). Furthermore, when Δ*cwlD* spores were purified, the levels of GerS were markedly reduced, whereas wild-type levels of GerS were present in Δ*pdaA* spores (Fig. 5B). Notably, complementation of *cwlD* restored signal peptide processing to GerS in sporulating cells and increased GerS levels in mature spores (Fig. 5B). All together, these results indicate that CwlD is critical for normal processing of GerS, leading to defects in GerS incorporation into or stability in mature spores or both.

In contrast with GerS, CwlD levels in sporulating cells were unchanged among the different mutant strains (Fig. 5B). However, Western blot analyses revealed that CwlD levels in mature spores appeared to be slightly (~2-fold) reduced in Δ*gerS* spores relative to wild-type spores, although this difference was not statistically significant (data not shown). CwlD also underwent cleavage in mature spores, with the cleaved variant of CwlD being more abundant than full-length CwlD in wild-type, Δ*gerS*, and Δ*pdaA* strains.

PdaA levels were unchanged in both sporulating cells and mature spores of wild-type and the mutant strains; both full-length and cleaved forms of PdaA were detected at equivalent levels in sporulating cells and mature spores in all the strains examined. Interestingly, although the B. subtilis homologs of PdaA and CwlD have predicted signal peptides, only PdaA has a putative signal peptide cleavage site, A_19_X_20_A_21_ (34, 37). In contrast, both C. difficile CwlD and PdaA lack predicted signal peptide sequences, although they both are predicted to contain transmembrane domains based on signal peptide 4.1 (52) and TMHMM v. 2.0 analyses. Since both full-length and cleaved CwlD and PdaA were detected by Western blotting, both proteins undergo some form of posttranslational processing, with processed CwlD being the predominant form detected in mature spores.

### GerS and CwlD physically interact.

**FIG 6:**
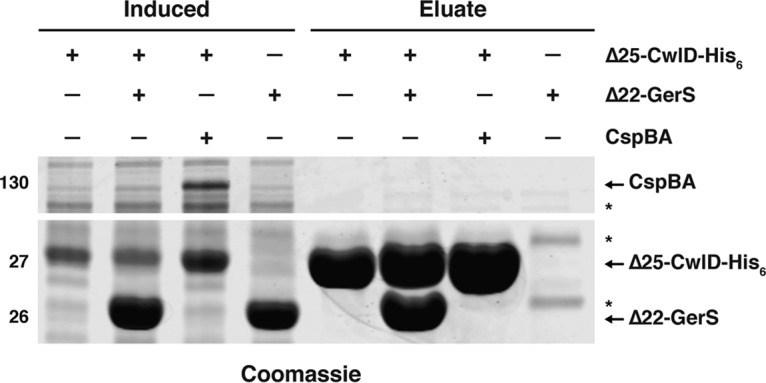
GerS and CwlD directly interact *in vitro*. (A) Coomassie stain of co-affinity purifications of His_6_-tagged CwlD_Δ25_ with empty vector, untagged CspBA, or untagged GerS. The indicated constructs were produced in E. coli following induction with IPTG and purified using Ni^2+^-affinity resin. IPTG-induced and eluate fractions were analyzed using Coomassie staining. Nonspecific bands are indicated by asterisks.

Since GerS processing in sporulating cells and GerS levels in spores were reduced in the absence of CwlD, we wondered whether CwlD might affect GerS processing through a direct interaction. To this end, we tested GerS binding to CwlD using co-affinity purification in an E. coli-based expression system. Purification of soluble CwlD and GerS from E. coli required deleting their transmembrane domain, so we deleted the region encoding CwlD’s putative transmembrane sequence and GerS’s signal peptide. C-terminally His-tagged CwlD lacking its N-terminal 25 residues (CwlD_Δ25_-His_6_) was coproduced with untagged GerS lacking its N-terminal 26 residues (GerS_Δ22_). As a specificity control, we also coproduced CwlD_Δ25_-His_6_ with untagged CspBA, which is also made in the mother cell during sporulation but was not expected to interact with CwlD. Notably, when CwlD_Δ25_-His_6_ was coproduced with untagged GerS in E. coli, untagged GerS was copurified with CwlD_Δ25_-His_6_ on Ni^2+^-affinity resin at stoichiometric levels (Fig. 6). In contrast, untagged CspBA did not copurify with CwlD_Δ25_-His_6_. Importantly, untagged GerS_Δ22_ exhibited little nonspecific binding to the Ni^2+^-affinity resin (Fig. 6). These results indicate that the soluble domains of recombinant GerS and CwlD directly interact with high affinity. They further suggest that GerS and CwlD directly interact in C. difficile sporulating cells, which may be related to GerS’s dependence on CwlD to undergo signal peptide processing.

## DISCUSSION

Although we previously showed that C. difficile spore germination depends on the GerS lipoprotein (20), the mechanism underlying this requirement remained unknown. In this study, we determined that GerS is necessary for CwlD-mediated cortex modification in C. difficile (Fig. 4) and that these cortex modifications are required for the SleC hydrolase to efficiently recognize its cortex substrate during germination (Fig. 1 and 3). Our results further revealed that GerS and CwlD directly interact (Fig. 6) and that GerS incorporation into spores depends on CwlD (Fig. 5). Since GerS homologs are found only in the members of the *Peptostreptococcaceae* family (20), our results suggest that C. difficile employs a unique mechanism for cortex modification relative to the pathway defined in B. subtilis (29) and possibly most other spore formers based on gene conservation.

Although the muropeptide profiles of C. difficile Δ*cwlD* and Δ*pdaA* suggest that C. difficile CwlD and PdaA have activities similar to those of their B. subtilis homologs, our studies raise the issue of why C. difficile CwlD’s presumed amidase activity depends on GerS, since CwlD in C. difficile and CwlD in B. subtilis share high amino acid similarity (58%). One possibility is that C. difficile CwlD amidase activity requires heterodimer formation with GerS, in contrast with B. subtilis CwlD, which functions independently (35). *In vitro* analyses of the peptidoglycan-modifying activities of recombinant CwlD and GerS, singly and in combination, would shed light on the ability of CwlD and/or the GerS-CwlD complex to modify peptidoglycan to generate NAM substrates for PdaA.

In addition to the interdependence between CwlD and GerS in modifying the cortex, CwlD regulates GerS signal peptide processing and incorporation into spores (Fig. 5). It is unclear whether the GerS processing defect in Δ*cwlD* spores alters GerS function, but mutation of GerS’s cysteine 22 lipidation site (20) in the presence of CwlD prevents signal peptide processing without altering GerS function or incorporation into spores (20). The GerS signal peptide processing defect of Δ*cwlD* spores may reflect the need for GerS to bind CwlD in order to be recognized by the C. difficile lipoprotein signal peptidase (Lsp [51]). Alternatively, CwlD-mediated stabilization of GerS after its transport across the outer forespore membrane or CwlD-dependent transport of GerS across this outer membrane could account for reduced GerS levels in Δ*cwlD* spores. Distinguishing between these possibilities requires that methods be developed for localizing proteins to the intermembrane space between the outer and inner forespore membranes. Unfortunately, current biochemical methods for removing coat layers disrupt the outer forespore membranes and strip cortex proteins from the spore (20).

Interestingly, both C. difficile CwlD and PdaA were detected in mature spores in both cleaved and uncleaved states, even though both proteins lacked any putative signal peptide cleavage site (Fig. 5), unlike their B. subtilis counterparts (34, 37, 52). While it is unclear whether cleaved C. difficile CwlD and PdaA are functional, their release from the outer and inner forespore membranes, respectively, could allow them to more efficiently modify cortex peptidoglycan, since their transcriptional regulation indicates they are produced in separate compartments. C. difficile *cwlD*, like *gerS*, is controlled by the mother cell-specific SigE sigma factor, while *pdaA* is controlled by the forespore-specific SigG sigma factor (53–55). The regulation of C. difficile *cwlD* contrasts slightly with that of B. subtilis, where *cwlD* is transcribed in both the mother cell and forespore (35, 37). Given that C. difficile CwlD appears to be released from the outer forespore membrane into the intermembrane space, whereas lipidation of GerS presumably retains GerS on the membrane, it is unclear whether cleaved CwlD remains associated with GerS on the outer forespore membrane. Regardless, the observation that GerS processing and incorporation into spores depend on CwlD (Fig. 5), CwlD’s presumed amidase activity depends on GerS (Fig. 4), and GerS and CwlD bind stoichiometrically in E. coli (Fig. 6) strongly suggests that GerS function and CwlD function are intimately related.

Indeed, the phenotypes of *gerS* and *cwlD* mutants, from their germination defects to their muropeptide profiles, were almost entirely overlapping (Fig. 1, 3, and 4). The *pdaA* mutant was also defective in generating cortex-specific muramic-δ-lactam (MAL) modifications and exhibited spore germination defects and heat sensitivity comparable to those seen with Δ*gerS* and *ΔcwlD* spores, even though the Δ*pdaA* spores produced a muropeptide profile that was slightly different from the profiles of the Δ*gerS* and Δ*cwlD* spores (Fig. 4). Taken together, our results indicate that C. difficile cortex modification is critical for spore germination and affects the apparent heat sensitivity of purified spores.

Notably, although the germination phenotypes of Δ*gerS* and Δ*cwlD* spores were indistinguishable from each other, C. difficile Δ*cwlD* exhibited circular rather than ovoid spore morphology three times more frequently than Δ*gerS* or wild-type cells (TEM, Fig. 2). It is unclear why loss of CwlD but not GerS altered spore morphology given that these strains give rise to spores with identical muropeptide profiles; indeed, alterations to spore morphology in B. subtilis *cwlD* mutant spores have not been mentioned in the literature (33, 56). Accordingly, it would be interesting to test whether CwlD’s ability to modify the cortex and regulate spore morphology depends on its enzymatic activity.

Taken together, our results confirm that the highly conserved CwlD and PdaA enzymes play roles in modifying the cortex similar to those played by their B. subtilis counterparts while also revealing a novel role for the GerS lipoprotein in regulating this process. The tight interaction between CwlD and GerS, their largely overlapping functions, and the conservation of GerS exclusively in the *Peptostreptococcaceae* family suggest that the mechanism underlying CwlD-mediated cortex modification may exhibit significant differences in C. difficile relative to B. subtilis and other spore-forming bacteria. Since we previously showed that GerS is required for virulence in a hamster model of C. difficile infection (20), the germination defects of *cwlD* and *pdaA* cortex modification mutants will likely reduce lethality in animal models of infection and reduce C. difficile disease recurrence. These analyses suggest that inhibiting GerS function during spore formation could reduce infectious C. difficile spore formation and thus disease transmission.

## MATERIALS AND METHODS

### Bacterial strains and growth conditions.

The 630Δ*erm ΔpyrE* parental strain was used for *pyrE*-based allelic coupled exchange (ACE [38]). The C. difficile strains used are listed in Table S2 in the supplemental material; they were grown on brain heart infusion-supplemented media (BHIS) supplemented with taurocholate (TA) (0.1% [wt/vol]; 1.9 mM), kanamycin (50 µg/ml), cefoxitin (8 µg/ml), FeSO_4_ (50 µM), and/or erythromycin (10 µg/ml) as needed. For ACE, C. difficile defined medium (CDDM) (57) was supplemented with 5-fluoroorotic acid (5-FOA) at 2 mg/ml and uracil at 5 µg/ml. Cultures were grown at 37°C under anaerobic conditions using a gas mixture containing 85% N_2_, 5% CO_2_, and 10% H_2_.

10.1128/mSphere.00205-18.9 TABLE S2  C. difficile and E. coli strains used in this study. Download TABLE S2, PDF file, 0.1 MB. Copyright © 2018 Diaz et al. 2018 Diaz et al. This content is distributed under the terms of the Creative Commons Attribution 4.0 International license.

The Escherichia coli strains used for HB101/pRK24-based conjugations and BL21(DE3)-based protein production are listed in Table S2. E. coli strains were grown at 37°C with shaking at 225 rpm in Luria-Bertani broth (LB). The medium was supplemented with chloramphenicol (20 µg/ml), ampicillin (50 µg/ml), or kanamycin (30 µg/ml) as indicated.

### E. coli strain construction.

All primers are listed in Table S3. All plasmid constructs were cloned into DH5α, and all sequences were confirmed using Genewiz. C. difficile 630 genomic DNA was used as the template, with the exception of the *cspBA* expression construct. To clone the pMTL-YN3-Δ*gerS* construct, primer pair 2007 and 2006 and primer pair 2005 and 2008 were used to amplify the regions 1,082 bp upstream and 987 bp downstream of *gerS*, respectively. The resulting PCR products were cloned into pMTL-YN3 using Gibson assembly (58). This construct results in an in-frame deletion of *gerS* where the first 12 codons are linked to the last 11 codons. To construct the *cwlD* mutant, primer pair 2389 and 2391 and primer pair 2390 and 2392 were used to amplify the regions 773 bp upstream and 728 bp downstream of *cwlD*. The resulting PCR products were used in a splicing by overhang extension PCR (SOE PCR) (59) with flanking primers 2389 and 2392 to generate a fragment with an in-frame deletion of *cwlD* where the first 21 codons are linked to the last 28 codons. To construct the *pdaA* mutant, primer pair 2430 and 2432 and primer pair 2431 and 2433 were used to amplify the regions 765 bp upstream and 849 bp downstream of *pdaA*. The resulting PCR products were used in a SOE PCR with flanking primers 2430 and 2433 to generate a fragment with an in-frame deletion of *cwlD* where the first 24 codons are linked to the last 25 codons. The resulting PCR products were recombined into pMTL-YN3 by Gibson assembly. The plasmids were transformed into E. coli DH5α, and the resulting plasmids were confirmed by sequencing and then transformed into HB101/pRK24.

10.1128/mSphere.00205-18.10 TABLE S3  Primers used in this study. Download TABLE S3, PDF file, 0.1 MB. Copyright © 2018 Diaz et al. 2018 Diaz et al. This content is distributed under the terms of the Creative Commons Attribution 4.0 International license.

To create the gerS* complementation construct, primer pair 2181 and 2182 was used to amplify *gerS*, including 387 bp of its upstream region. To construct the *gerS-alr2* complementation construct, primer pair 2181 and 2408 was used to amplify 387 bp upstream and downstream of the *alr2* gene. While both the *gerS* and *gerS-alr2* complementation constructs restored wild-type levels of germination to *ΔgerS* (see Fig. S7 in the supplemental material), GerS levels were consistently higher in the *gerS-alr2* complementation construct than in the *gerS* construct. To create the *cwlD* complementation construct, primer pair 2362 and 2450 was used to amplify *cwlD* and 274 bp of its upstream region. To create the *pdaA* complementation construct, primer pair 2451 and 2457 was used to amplify *pdaA* and 198 bp upstream and 138 bp downstream of the *pdaA* gene. The resulting PCR products were recombined into pMTL-YN1C by Gibson assembly. The plasmids were transformed into E. coli DH5α, and the resulting plasmids were confirmed by sequencing and then transformed into HB101/pRK24.

10.1128/mSphere.00205-18.7 FIG S7  Optimization of Δ*gerS* complementation constructs. (A) Western blot analysis of GerS in Δ*gerS* complementation strains. CotA was used as a loading control. (B) Germinated spore CFU generated from *gerS* complementation strains plated on BHIS-TA. Download FIG S7, TIF file, 0.4 MB. Copyright © 2018 Diaz et al. 2018 Diaz et al. This content is distributed under the terms of the Creative Commons Attribution 4.0 International license.

To create the recombinant protein expression constructs for co-affinity purifications and antibody production, primer pair 2545 and 2546 was used to amplify *cwlD* lacking its first 25 codons, which encode the transmembrane domain, and the stop codon, using C. difficile genomic DNA as the template. The resulting PCR product was cloned into pET22b to result in a CwlD_Δ25_-His_6_ fusion construct using Gibson assembly. The resulting plasmids were confirmed by sequencing and then transformed into BL21(DE3) cells.

To create the *pdaA* expression construct for antibody production, primer pair 1458 and 1459 was used to amplify full-length *pdaA* without its stop codon. The resulting PCR product was digested with NdeI and XhoI and cloned into pET22b digested with the same enzymes to generate a construct encoding C-terminally His_6_-tagged PdaA. The *cspBA* expression construct was cloned using a series of primers and plasmids pKS01 and pKS02 as the template, which harbor codon-optimized *cspA* and *cspB*, respectively, and were a kind gift of Joseph Sorg. Primer pair 1505 and 1529 was first used for PCR analysis of codon-optimized *cspB*. Primer pair 1507 and 1508 was used for PCR analysis of codon-optimized *cspA* lacking its stop codon. The resulting PCR template was used in a second PCR with primer pair 1530 and 1508 to extend the 5′ end of the PCR product to overlap the codon-optimized *cspB* PCR. The resulting PCR product was used in a SOE PCR reaction with the *cspB* template to generate codon-optimized *cspBA* lacking its stop codon. This product was cloned into pET28a by digesting both with NcoI and XhoI and ligating the products. The resulting plasmid, pET28a-*cspBA*, was then used as the template with primer pair 1505 and 1625. The resulting PCR product harbors codon-optimized *cspBA* with a stop codon; this product was gel purified and then digested with NcoI and XhoI and ligated into pET28a cut with the same enzymes.

### Bioinformatic analyses.

Homologs of C. difficile 630 CwlD (CD630_01060) and PdaA (CD630_14300) were identified using NCBI PSI-BLAST. Homologs identified in *Peptostreptococcaceae* family members gave an E value of <e^−124^, in *Clostridium* spp. an E value of <e^−48^, in *Bacillus* spp. an E value of <e^−49^, and in *Paenibacillus* spp. an E value of <e^−62^. Both signal peptide cleavage sites (SignalP 4.1 [52]) and transmembrane helices (TMHMM v. 2.0 [60]) were predicted for C. difficile GerS, CwlD, and PdaA.

### C. difficile strain construction.

Allele-coupled exchange (ACE [38]) was used to construct clean deletions of *gerS*, *cwlD*, and *pdaA* in the 630Δ*erm* Δ*pyrE* mutant using uracil and 5-fluoroorotic acid to select for plasmid excision as previously described (44). Flanking primers used to screen for deletions of *gerS*, *cwlD*, and *pdaA* are shown in Fig. S2 and provided in Table S3. Colonies that appeared to harbor gene deletions were confirmed using a primer that binds within the region with a primer that binds to the flanking region as a negative control. At least two independent clones from each allelic exchange were phenotypically characterized.

Complementation strains were constructed as previously described using CDDM to select for restoration of the *pyrE* locus via recombination of the complementation construct into that locus (44). Two independent clones from each complementation strain were phenotypically characterized.

### Sporulation.

C. difficile strains were inoculated from glycerol stocks overnight onto BHIS plates containing taurocholate (TA) (0.1% [wt/vol], 1.9 mM). Colonies arising from these plates were used to inoculate liquid BHIS cultures, which were grown to early stationary phase, back-diluted 1:50 into BHIS, and grown until the cultures reached an OD_600_ between 0.35 and 0.75. Sporulation was induced on 70:30 agar plates as previously described (45) for 24 h. Sporulating cells were harvested into phosphate-buffered saline (PBS), and sporulation levels were visualized by phase-contrast microscopy.

### Phase-contrast microscopy.

Sporulating cultures were enumerated for visible signs of sporulation using phase-contrast microscopy. A minimum of 450 cells from three independent replicates were counted, and the percentage of circular forespore and free spore formation was determined relative to the total number of cells with visible signs of sporulation combined with the number of free spores. The cell counts were averaged, and the standard deviation was calculated. Statistical significance was determined using a one-way analysis of variance (ANOVA) and Tukey’s test.

### Sporulation efficiency assay.

Heat-resistant, functional spore formation was measured in sporulating C. difficile cultures after 20 to 24 h as previously described (40). The apparent sporulation efficiency represents the average ratio of heat-resistant CFU to total CFU for a given strain relative to the average ratio determined for the wild type based on a minimum of three biological replicates. Statistical significance was determined using a one-way ANOVA and Tukey’s test.

### Spore purification.

Sporulation was induced on 70:30 agar plates for 2 to 3 days as previously described (45). Spores were washed 6 times in ice-cold water, incubated overnight in water on ice, treated with DNase I (New England Biolabs) at 37°C for 60 min, and purified on a HistoDenz (Sigma-Aldrich) gradient. Phase-contrast microscopy was used to assess spore purity (>95% pure); the optical density of the spore stock was measured at OD_600_; and spores were stored in water at 4°C. Despite the circular spore morphology, spore purification yields for the *cwlD* and *pdaA* mutants were comparable to those of the *gerS* mutant and wild-type spores (Fig. 1C). Spore purification yields were determined from four biological replicates performed using two independent spore preparations. Four 70:30 plates were used to induce sporulation for each strain; purified spores were resuspended in 600 µl, and the total optical density of this mixture was determined for each strain.

### Spore heat sensitivity.

To assess spore heat sensitivity, ~2 × 10^7^ spores were resuspended in 510 µl of water (OD_600_ of 1.6). The solution was aliquoted as 100-µl volumes into two tubes. A 10-µl volume was serially diluted for the untreated condition. The remaining four tubes were incubated for 15 min at room temperature or 60°C for 30 min. Samples were serially diluted in PBS following heat treatment and plated onto BHIS-TA. After ~23 h, colonies arising from germinated spores were counted. The remaining 90 µl of the spores was pelleted and resuspended in EBB buffer (8 M urea, 2 M thiourea, 4% [wt/vol] SDS, 2% [vol/vol] β-mercaptoethanol) for Western blot analyses (see below).

### Germination assay.

Germination assays were performed as previously described (20). Approximately 1 × 10^7^ spores (OD_600_ of 0.35) were resuspended in 100 µl of water, and 10 µl of this mixture was removed for 10-fold serial dilutions in PBS. The dilutions were plated on BHIS-TA, and colonies arising from germinated spores were enumerated at 23 h. Germination efficiencies were calculated by averaging the CFU produced by spores for a given strain relative to the number produced by wild-type spores for at least three biological replicates from two independent spore preparations. Statistical significance was determined by performing a one-way analysis of variance (ANOVA) on natural log-transformed data using Tukey’s test. The data were transformed because the use of two independent spore preparations resulted in a nonnormal distribution.

### OD_600_ kinetics assay.

Germination was induced as previously described (44). About 1.4 × 10^7^ spores (OD_600_ of 0.48) were suspended in BHIS and exposed to 1% taurocholate (19 mM), and the OD_600_ was measured every 3 min for 45 min, followed by every 15 min for a total of 3 h. The change in OD_600_ over time was determined by calculating the ratio of the OD_600_ measured for TA-treated samples to that measured for untreated samples. Statistical significance was measured by performing a repeated-measures ANOVA and Tukey’s test.

### Total DPA quantification using fluorescence.

To evaluate the total amount of DPA contained within spores using terbium fluorescence, approximately 2 × 10^7^ spores from each strain were resuspended in 1 ml of buffer 1 [0.3 mM (NH_4_)_2_SO_4_, 6.6 mM KH_2_PO_4_, 15 mM NaCl, 59.5 mM NaHCO_3_, and 35.2 mM Na_2_HPO_4_] and incubated at 37°C (background) or 95°C (total DPA) for 1 h. Following heat treatment, 10 µl of supernatant was added to 115 µl of buffer 2 (1 mM Tris, 150 mM NaCl) with 200 µM terbium chloride (Alfa Aesar). Samples were prepared in an opaque 96-well plate (PerkinElmer) and evaluated after 15 min of incubation with terbium chloride using a Synergy H1 microplate reader (BioTek; 270-nm excitation, 420 nm cutoff, 545-nm reading, gain of 100). Reported relative fluorescent unit (RFU) values represent the background fluorescence (wild-type, Δ*gerS*, Δ*cwlD*, Δ*pdaA*, and Δ*sleC* spores, incubated at 37°C, with terbium) and were subtracted from the fluorescence detected for each strain after 95°C treatment. The data represent results from three biological replicates.

### Transmission electron microscopy.

Sporulating cultures (23 h) were fixed and processed for electron microscopy at the University of Vermont Microscopy Imaging Center as previously described (61). A minimum of 50 full-length sporulating cells were used for phenotype counting, with the exception that 25 full-length sporulating cells were counted to analyze the phenotype of the *cwlD* complementation strain.

### Muropeptide analysis.

Purified spores were used for cortex peptidoglycan purification, muramidase digestion, and HPLC separation of muropeptides essentially as described previously (30). Peaks were collected, further purified by HPLC using a volatile buffer system, and subjected to amino acid analysis as described previously (62) using matrix-assisted laser desorption ionization–time of flight mass spectrometry (MALDI-TOF MS) to analyze muropeptides as described previously (63). Briefly, muropeptides were dissolved in HPLC-grade water and 1 µl of each sample was spotted and dried on a MALDI plate. Muropeptide spots were covered with 1 µl of 4 mg/ml α-cyano-4-hydroxycinnamic acid–50% (vol/vol) acetonitrile–0.2% (vol/vol) trifluoroacetic acid–20-mM ammonium chloride and air dried. Mass spectra were acquired using a 4800 MALDI-TOF/TOF mass spectrometer (Applied Biosystems) in positive ion mode with a mass-to-charge range of 500 to 2,000 using the average from 1,500 individual laser shots. Parent ions were further fragmented utilizing tandem mass spectrometry (MS/MS) in 1-kV positive operating mode, and tandem mass spectra were generated from the sum of 2,500 individual laser shots.

### Western blot analysis.

Samples for immunoblotting were prepared as previously described (61). Briefly, sporulating cell pellets were resuspended in 100 µl of PBS, and 50-µl samples were freeze-thawed for three cycles and then resuspended in 100 µl EBB buffer (8 M urea, 2 M thiourea, 4% [wt/vol] SDS, 2% [vol/vol] β-mercaptoethanol). C. difficile spores (~1 × 10^6^) were resuspended in EBB buffer, which can extract proteins in all layers of the spore, including the core. Both sporulating cells and spores were incubated at 95°C for 20 min with vortex mixing. Samples were centrifuged for 5 min at 15,000 rpm, and 4× sample buffer (40% [vol/vol] glycerol, 1 M Tris [pH 6.8], 20% [vol/vol] β-mercaptoethanol, 8% [wt/vol] SDS, 0.04% [wt/vol] bromophenol blue) was added. Samples were incubated again at 95°C for 5 to 10 min with vortex mixing followed by centrifugation for 5 min at 15,000 rpm. The samples were resolved by the use of 12% SDS-PAGE gels and transferred to a Millipore Immobilon-FL polyvinylidene difluoride (PVDF) membrane. The membranes were blocked in Odyssey blocking buffer with 0.1% (vol/vol) Tween 20. Polyclonal rabbit anti-GerS (20), anti-CwlD, anti-PdaA, anti-CspB, and anti-CotA (25) antibodies were used at a 1:1,000 dilution. Polyclonal mouse anti-Spo0A (64) was used at 1:1,000 dilutions, and mouse anti-SleC (42) antibody was used at a 1:5,000 dilution. IRDye 680CW and 800CW infrared dye-conjugated secondary antibodies were used at 1:20,000 dilutions. Odyssey LiCor CLx was used to detect secondary antibody infrared fluorescence emissions.

### SleC cleavage assay.

Germinant-induced processing of the pro-SleC zymogen was assessed using ~2 × 10^7^ spores per strain suspended in samples containing 100 µl of water and 100 µl of BHIS. The spore solution was aliquoted in 90-µl volumes into two tubes containing either 10 µl of water or 10 µl of a 10% taurocholate (19 mM) solution. After incubation for 20 min at 37°C, the samples were pelleted for 5 min at 15,000 rpm, 50 µl of EBB buffer was added, and samples were prepared for Western blot analysis as described above.

### Protein purification for antibody production and His_6_ tag pulldown assays.

E. coli BL21(DE3) strains (see Table S2 in the supplemental material) were grown for protein purification. The anti-CwlD and anti-PdaA antibodies used in this study were raised against CwlD_Δ25_-His_6_, which lacks its first 25 codons, and against full-length PdaA-His_6_ in rabbits by Cocalico Biologicals (Reamstown, PA). Δ25-CwlD-His_6_ was purified from E. coli strains 2045 using Ni^2+^-affinity resin as described in more detail below. PdaA-His_6_ was purified similarly as well except that an additional gel filtration chromatography step was used.

Cultures were grown to mid-log phase in 2YT (5 g NaCl, 10 g yeast extract, 15 g tryptone per liter), induced with 250 µM isopropyl-β-d-1-thiogalactopyranoside (IPTG), and grown overnight at 19°C. Cultures were then pelleted, resuspended in lysis buffer (500 mM NaCl, 50 mM Tris [pH 7.5], 15 mM imidazole, 10% [vol/vol] glycerol), flash frozen in liquid nitrogen, and sonicated. The insoluble material was pelleted, and the soluble fraction was incubated with nickel-nitrilotriacetic acid (Ni-NTA) agarose beads (5 Prime) (0.5 ml) for 3 h and eluted using high-imidazole buffer (500 mM NaCl, 50 mM Tris [pH 7.5], 200 mM imidazole, 10% [vol/vol] glycerol) after nutating the sample for 5 to 10 min. The resulting induction and eluate fractions were run on 12% SDS-PAGE gels and stained using GelCode Blue colloidal blue total protein stain (Pierce) or transferred onto a PVDF membrane for Western blot analysis, as described above.

For the co-affinity purification assays, E. coli BL21(DE3) strains were transformed with two plasmids to express combinations of Δ25-CwlD-His_6_ or untagged Δ22-GerS or untagged CspBA or empty vector and purified using Ni^2+^-affinity resin as described above.
