# Supplementary figures and images for: Clostridium difficile Lipoprotein GerS Is Required for Cortex Modification and Thus Spore Germination
Source: mSphere. 2018 Jun 27;3(3):e00205-18. doi: 10.1128/mSphere.00205-18 (PMC6021603; doi:10.1128/mSphere.00205-18)

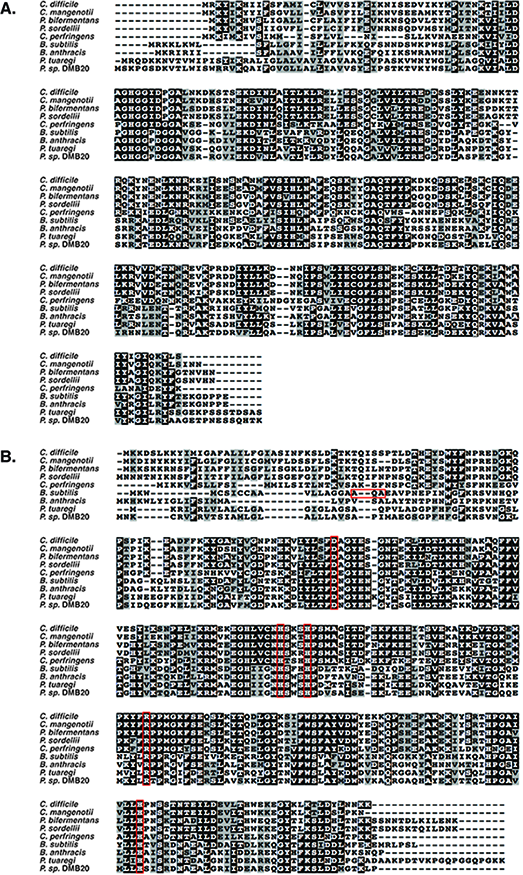

Supplement: FIG S1 [file sph003182575sf1.tif]

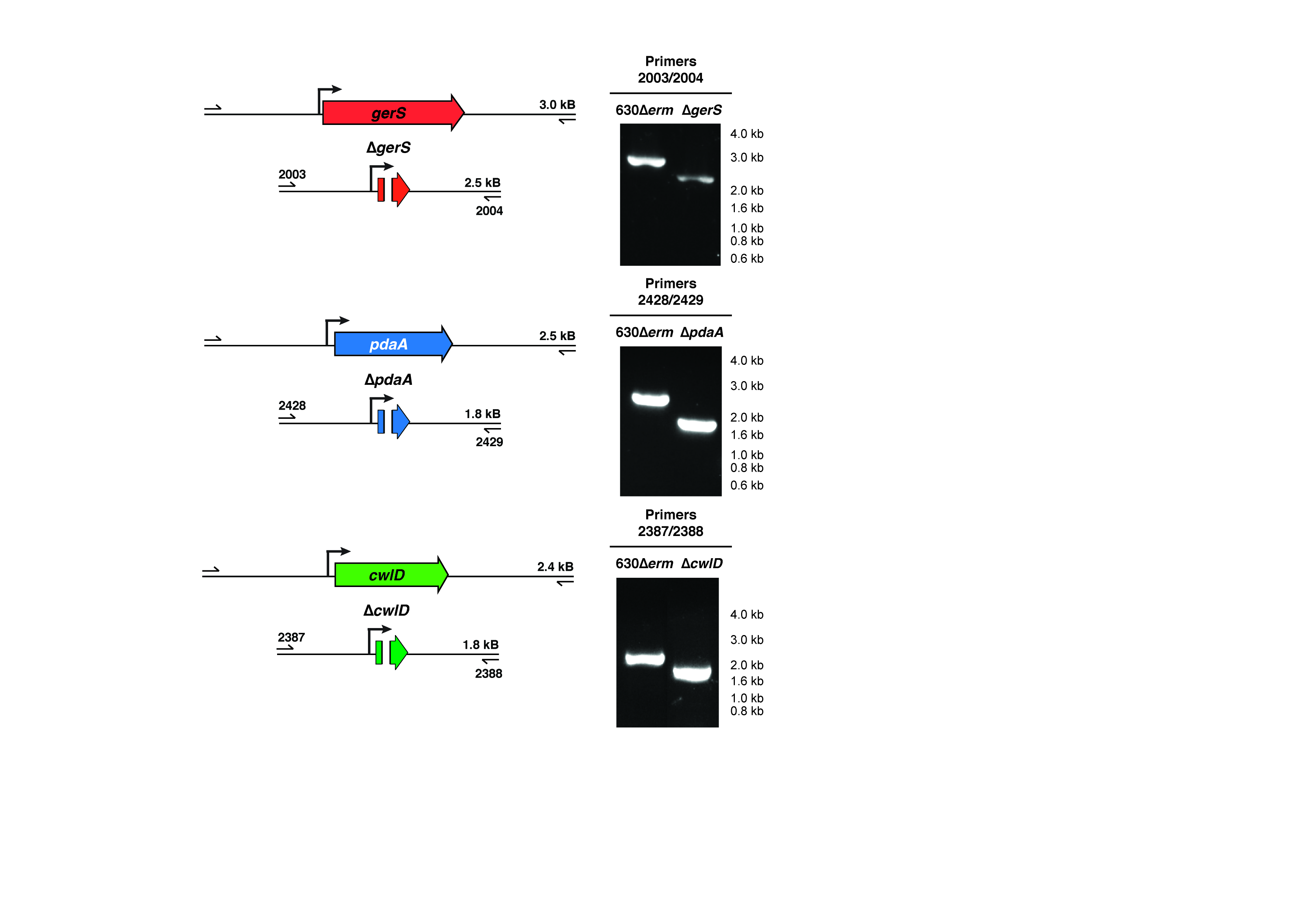

Supplement: FIG S2 [file sph003182575sf2.tif]

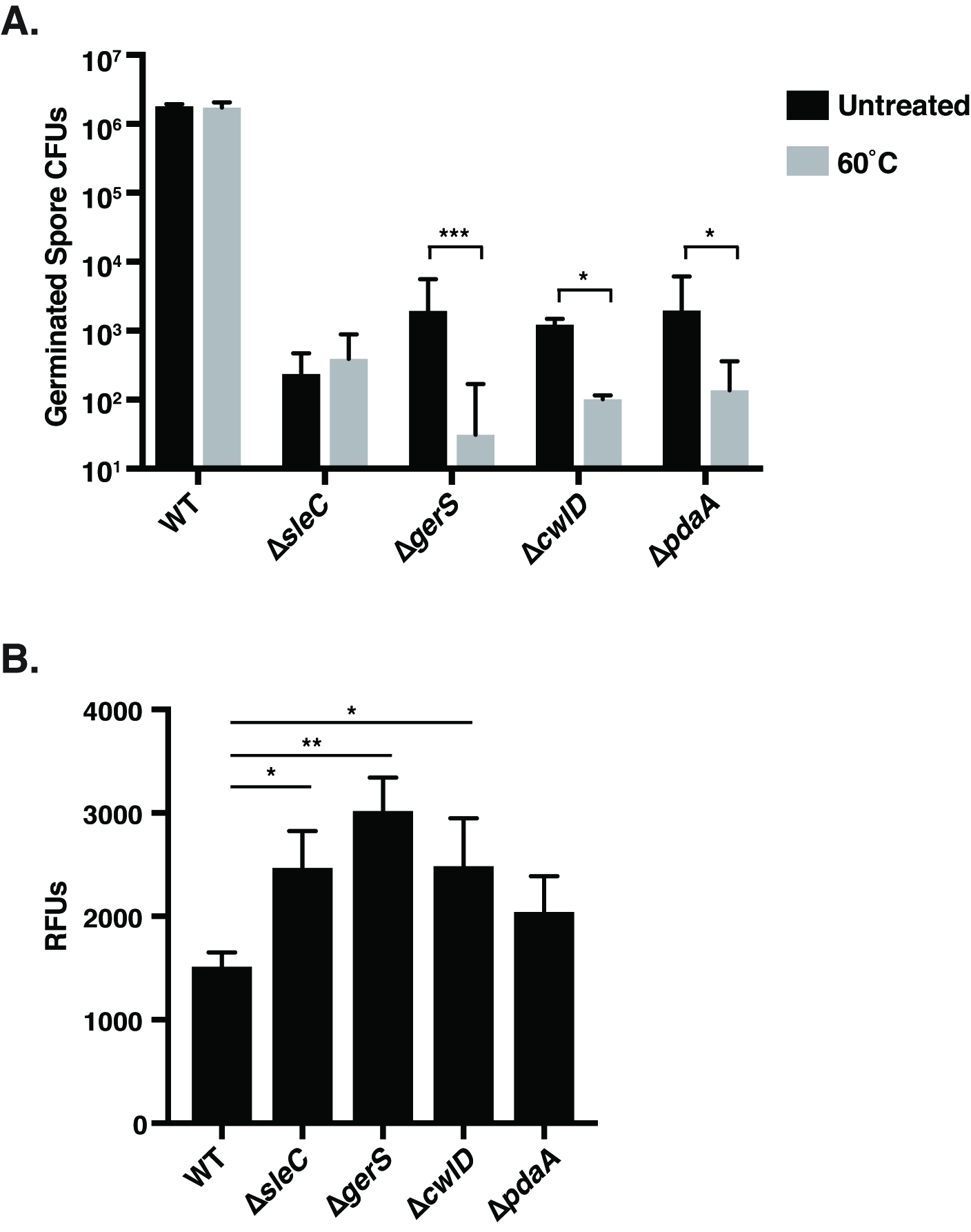

Supplement: FIG S3 [file sph003182575sf3.tif]

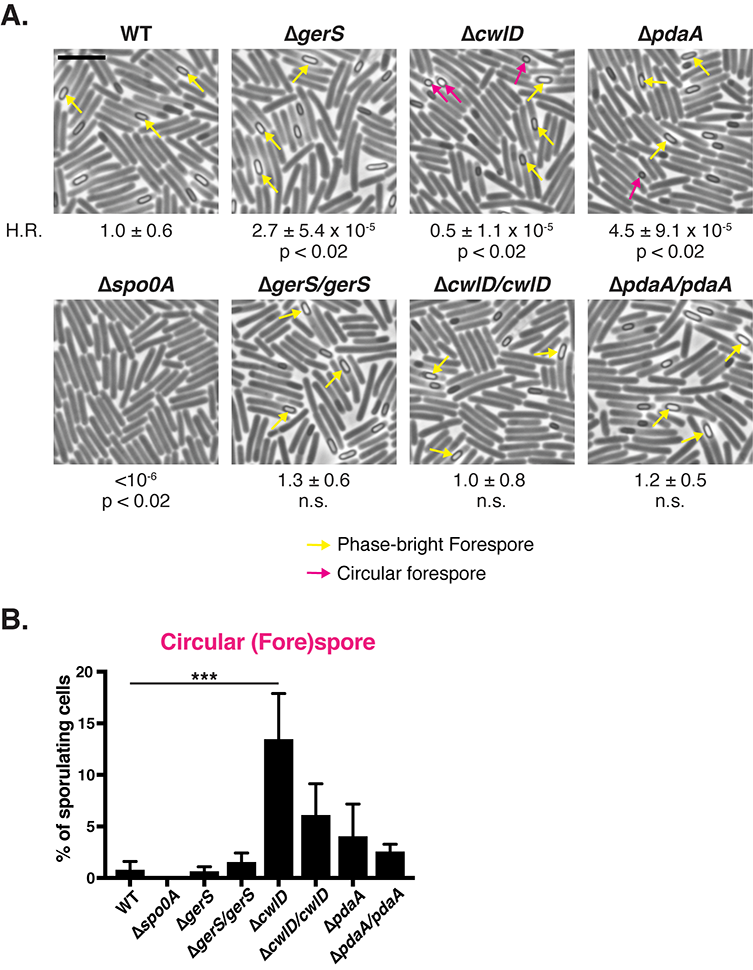

Supplement: FIG S4 [file sph003182575sf4.tif]

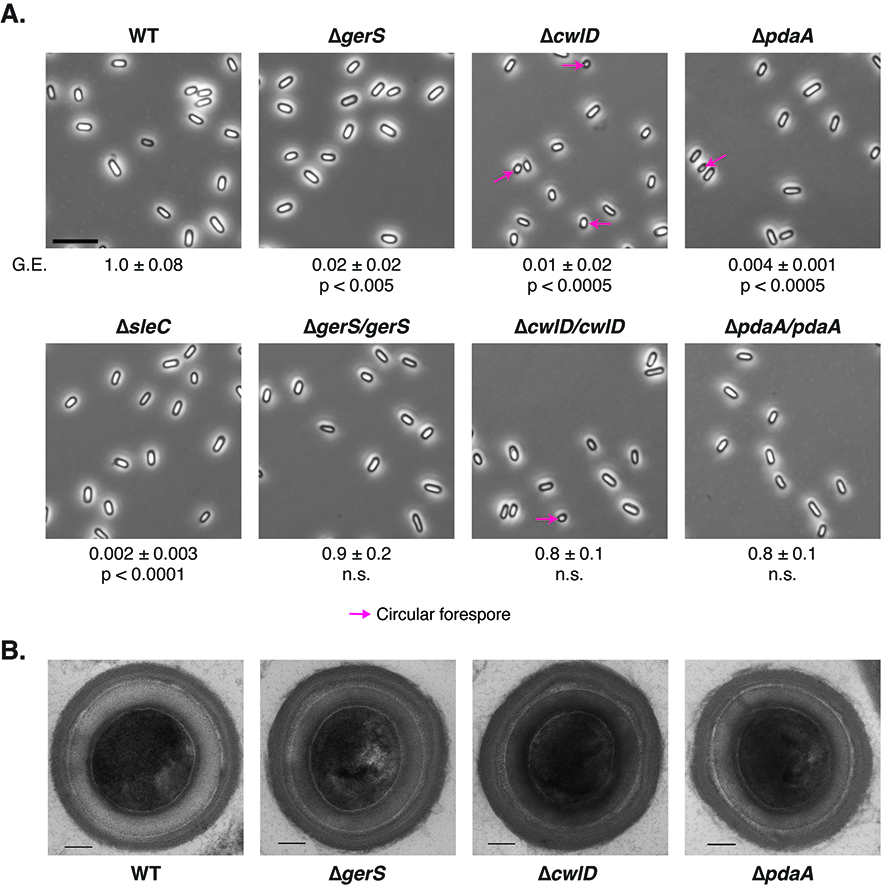

Supplement: FIG S5 [file sph003182575sf5.tif]

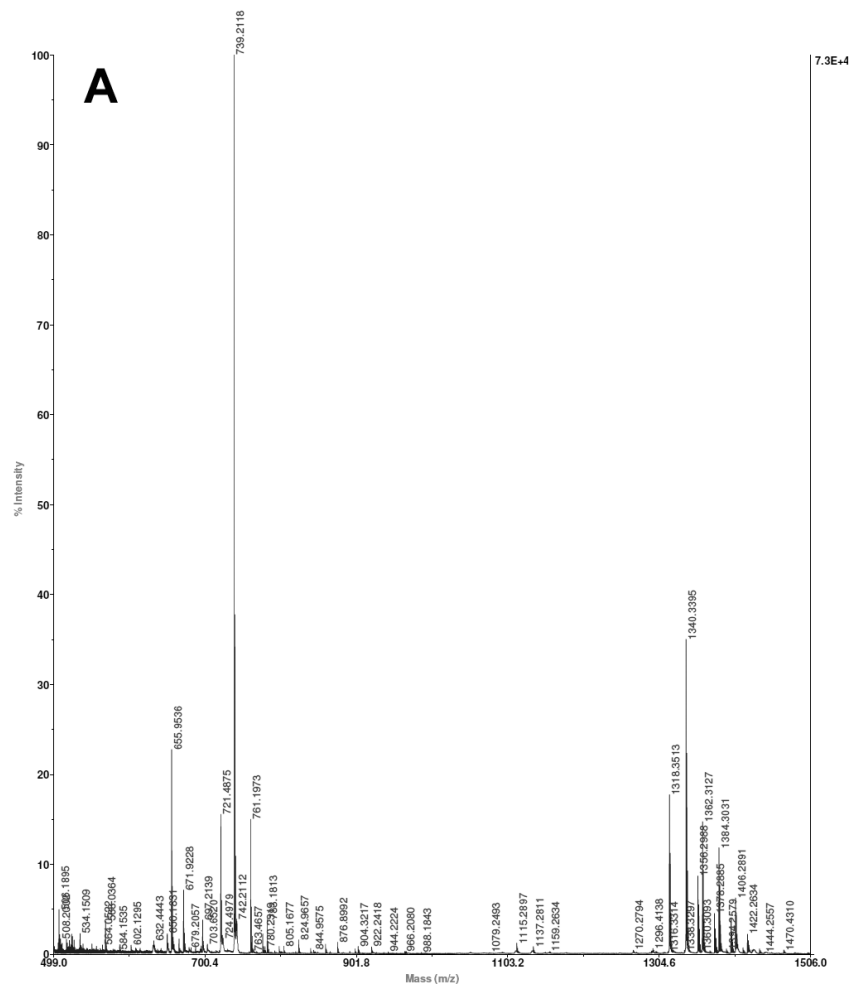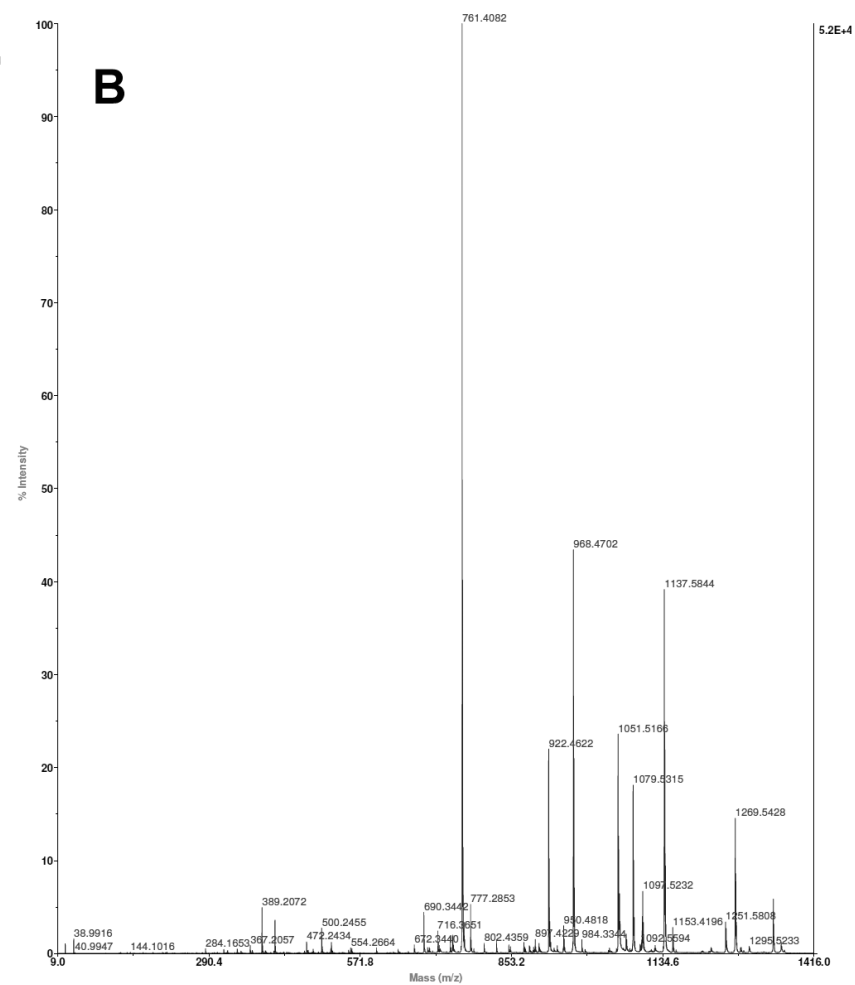

Supplement: FIG S6 [file sph003182575sf6.pdf]

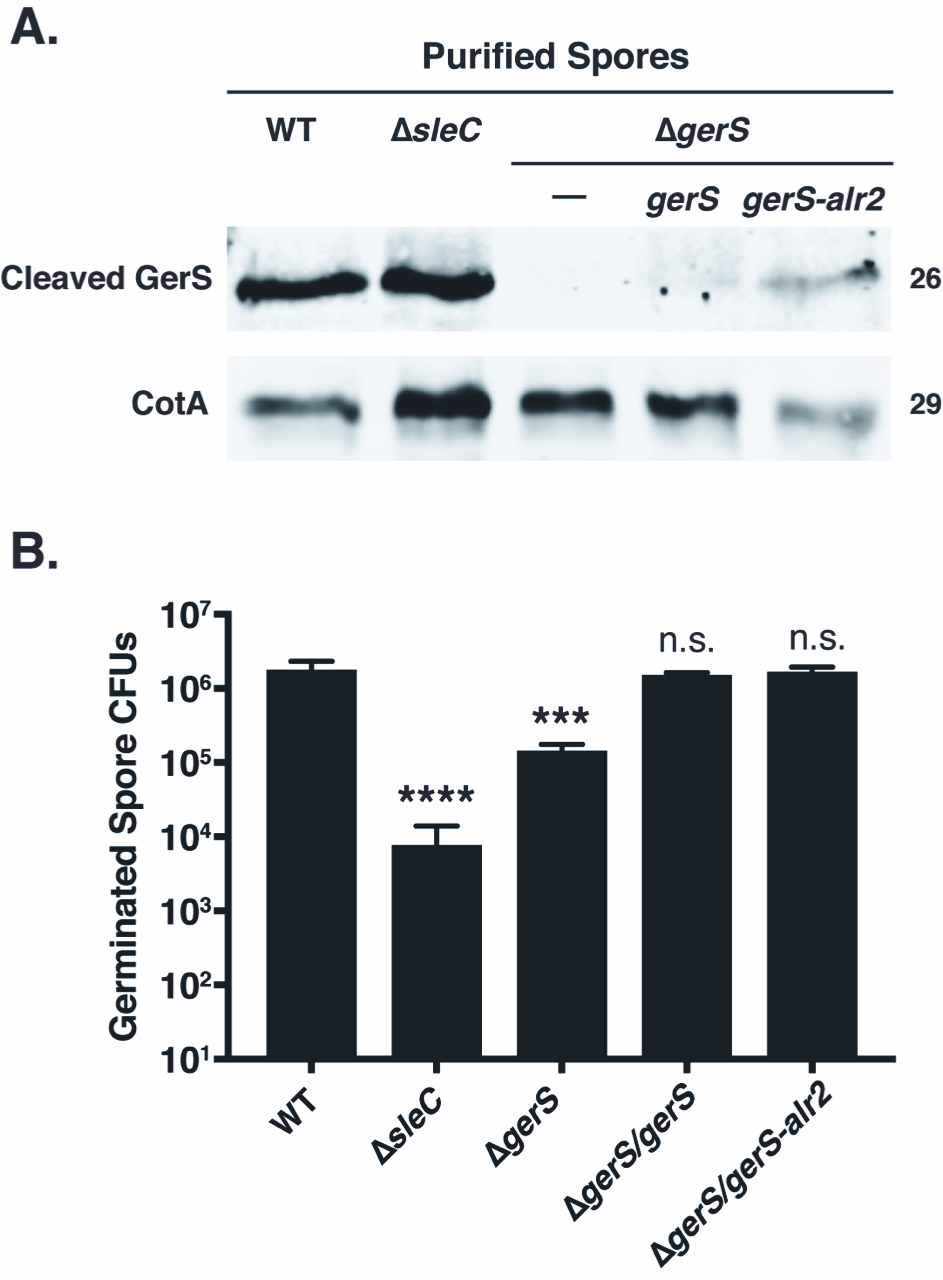

Supplement: FIG S7 [file sph003182575sf7.tif]
