# Supplementary material for: Clostridium difficile Lipoprotein GerS Is Required for Cortex Modification and Thus Spore Germination
Source: mSphere. 2018 Jun 27;3(3):e00205-18. doi: 10.1128/mSphere.00205-18 (PMC6021603; doi:10.1128/mSphere.00205-18)
Supplement: TABLE S1 [file sph003182575st1.pdf]

Table S1. MS-MS analyses of muropeptide structures

Peak	Parent ion Na^+ m/z	Muropeptide Structure	MS/MS Fragment Na^+ m/z	Fragment neutral loss	Fragment structure loss	Fragment structure remaining
**1**	999.2	NAG-NAM-NAG-NAMoh	927.4	71.8	Lactyl group of NAM	NAG-NAG-NAG-NAMoh
			796.4	202.8	NAG	NAM-NAG-NAMoh
			704.3	294.9	NAMoh and HO[1]	NAG-NAM-NAGanhydro[1]
			521.3	477.9	NAG-NAM	NAG-NAMoh
			501.3	497.9	NAG-NAMoh and HO[1]	NAG-NAManhydro[1]
**2**	964.3	DS-TP	893.2	71.1	Ala	DS-TriP
			761.2	203.1	NAG	NAMoh-TP
			592.2	372.1	Glu-Dpm-Ala	DS-Ala
**3**	922.3	DS-TP deacetylated	851.2	71.1	Ala	DS-TriP deacetylated
			761.2	161.1	GlucN	NAMoh-TP
			550.1	372.2	Glu-Dpm-Ala	DS-Ala deacetylated
**4**	721.2	DS-DiP	592.2	129	Glu	DS-Ala
			518.2	203	NAG	NAMoh-DiP
			389.2	332	NAG and Glu	NAMoh-Ala
**5**	679.2	DS-DiP deacetylated	550.2	129	Glu	DS-Ala deacetylated
			518.2	161	GlucN	NAMoh-DiP
			389.1	290.1	GlucN and Glu	NAMoh-Ala
**6**	1400.3	GlucN-NAM-NAG-NAMoh-TP	1328.5	71.8	Lactyl group of NAM	GlucN-NAG-NAG-NAMoh-TP
			1239.5	160.8	GlucN	NAM-NAG-NAMoh-TP
			1028.4	371.9	Glu-Dpm-Ala	GlucN-NAM-NAG-NAMoh-Ala
			964.4	435.9	GlucN-NAM	DS-TP
			761.4	638.9	GlucN-NAM-NAG	NAMoh-TP
			592.3	808	GlucN-NAM and Glu-Dpm-Ala	DS-Ala
**7**	1358.3	GlucN-NAM-GlucN-NAMoh-TP	1286.5	71.8	Lactyl group of NAM	GlucN-NAG-GlucN-NAMoh-TP
			1197.5	160.8	GlucN	NAM-GlucN-NAMoh-TP
			986.5	371.8	Glu-Dpm-Ala	GlucN-NAM-GlucN-NAMoh-Ala
			922.5	435.8	GlucN-NAM	DS-TP deacetylated
			761.4	596.9	GlucN-NAM-GlucN	NAMoh-TP
			550.3	808	GlucN-NAM and Glu-Dpm-Ala	DS-Ala deacetylated

**8**	1400.9	NAG-NAM-GlucN-NAMoh-TP	1328.3	72.6	Lactyl group of NAM	NAG-NAG-GlucN-NAMoh-TP
			1197.3	203.6	NAG	NAM-GlucN-NAMoh-TP
			1028.3	372.6	Glu-Dpm-Ala	NAG-NAM-GlucN-NAMoh-Ala
			922.3	478.6	NAG-NAM	GlucN-NAMoh-TP
			761.2	639.7	GlucN-NAM-NAG	NAMoh-TP
**9**	1887.7	DS-TP-TP-DS	1816.5	71.2	Ala	DS-TP-TriP-DS
			1684.5	203.2	NAG	NAMoh-TP-TP-DS
			1336.5	551.2	NAG-NAMoh-Ala -H_2O[2]	DS-TP-Ala-Dpm-Glu[2]
			1207.5	680.2	NAG-NAMoh-Ala-Glu -H_2O[2]	DS-TP-Ala-Dpm[2]
			964.4	923.3	DS-TP	DS-TP
			592.2	1295.5	DS-TP-TriP	DS-Ala
**10**	1845.7	DS-TP-TP-DS deacetylated	1774.3	71.4	Ala	DS-TP-TriP-DS deacetylated
			1684.5	161.2	GlucN	DS-TP-TP-NAMoh
			1642.5	203.2	NAG	NAMoh-TP-TP-DS deacetylated
			1481.5	364.2	GlucN and NAG	NAMoh-TP-TP-NAM
			1336.4	509.3	GlucN-NAMoh-Ala -H_2O[2]	DS-TP-Ala-Dpm-Glu[2]
			1294.4	551.3	DS-Ala -H_2O[2]	GlucN-NAMoh-TP-Ala-Dpm-Glu[2]
			1207.5	638.2	GlucN-NAMoh-Ala-Glu -H_2O[2]	DS-TP-Ala-Dpm[2]
			1165.5	680.2	DS-Ala-Glu -H_2O[2]	GlucN-NAMoh-TP-DiP[2]
			1004.4	841.3	DS-Ala-Glu -H_2O[2] -GlucN	NAMoh-TP-Ala-Dpm[2]
			964.4	881.3	GlucN-NAMoh-TP	DS-TP
			592.2	1253.5	DS-TP-TriP	DS-Ala
**11**	1845.7	DS-TP-TP-DS deacetylated	1774.5	71.2	Ala	DS-TP-TriP-DS deacetylated
			1684.5	161.2	GlucN	DS-TP-TP-NAMoh
			1642.5	203.2	NAG	NAMoh-TP-TP-DS deacetylated
			1481.5	364.2	GlucN and NAG	NAMoh-TP-TP-NAMoh
			1336.4	509.3	GlucN-NAMoh-Ala -H_2O[2]	DS-TP-Ala-Dpm-Glu[2]
			1294.4	551.3	NAG-NAMoh-Ala -H_2O[2]	GlucN-NAMoh-TP-Ala-Dpm-Glu[2]
			1207.4	638.3	GlucN-NAMoh-Ala-Glu -H_2O[2]	DS-TP-Ala-Dpm[2]
			1165.4	680.3	NAG-NAMoh-Ala-Glu -H_2O[2]	GlucN-NAMoh-TP-DiP[2]
			1004.4	841.3	NAG-NAMoh-Ala-Glu -H_2O[2] -GlucN	NAMoh-TP-DiP[2]
			964.4	881.3	GlucN-NAMoh-TP	DS-TP
			592.2	1253.5	DS-TP- Ala-Dpm-Glu	DS-Ala

**12**	1340.3	TS-TP deacetylated	1269.5	70.8	Ala	TS-TriP deacetylated
			1137.6	202.7	NAG	MAL-GlucN-NAMoh-TP
			968.5	371.8	Glu-Dpm-Ala	TS-Ala deacetylated
			761.4	578.9	NAG-MAL-GlucN	NAMoh-TP

1 Fragmentation to remove the reducing end sugar appears to result in the loss of OH and the formation of an anhydro sugar on the remaining ion.

2 Fragmentation within cross-linked peptides seems to often result in cyclization within the remaining peptide and loss of an H_2O.

3 Abbreviaitons: DS =NAG-NAMoh; TS = NAG-MAL-NAG-NAMoh; GlucN =Deacetylated NAG (glucosamine)(Deaccetylation is always on NAG); DiP = Ala-Glu; TriP = Ala-Glu-Dpm; TP = Ala-Glu-Dpm-Ala; NAM that has lost 71.8 Da lactyl group becomes NAG.
