# Supplementary material for: Clostridium difficile Lipoprotein GerS Is Required for Cortex Modification and Thus Spore Germination
Source: mSphere. 2018 Jun 27;3(3):e00205-18. doi: 10.1128/mSphere.00205-18 (PMC6021603; doi:10.1128/mSphere.00205-18)
Supplement: TABLE S2 [file sph003182575st2.pdf]

Table S2. Strains and plasmids used in this study

Strain #	Strain name	Relevant genotype or features	Source/reference
C. difficile* strains – 630Δ*erm			
756	630Δ*erm*Δ*pyrE*	*erm*-sensitive derivate of 630 with a deletion in *pyrE*	[1]
846	630Δ*erm*-p	*erm*-sensitive derivate of 630 with *pyrE* restored	[2]
849	630Δ*erm* Δ*spo0A*-p	630Δ*erm*Δ*spo0A* with *pyrE* restored	[2]
925	630Δ*erm* Δ*sleC*-p	630Δ*erm*Δ*sleC* with *pyrE* restored	[2]
950	630Δ*erm* Δ*gerS*Δ*pyrE*	630Δ*erm*Δ*pyrE* with *gerS* deleted (*CD630_34640*)	This study
1075	630Δ*erm* Δ*gerS*-p	630Δ*erm*Δ*gerS* with *pyrE* restored	This study
1084	630Δ*erm* Δ*gerS*/*gerS**	630Δ*erm*Δ*gerS* with *gerS* in the *pyrE* locus	[3]
1611	630Δ*erm* Δ*pdaA*Δ*pyrE*	630Δ*erm*Δ*pyrE* with *pdaA* deleted (*CD630_14300)*	This study
1617	630Δ*erm* Δ*cwlD*Δ*pyrE*	630Δ*erm*Δ*pyrE* with *cwlD* deleted (*CD630_01060*)	This study
1677	630Δ*erm* Δ*gerS*/*gerS*	630Δ*erm*Δ*gerS* with *gerS-alr2* in the *pyrE* locus	This study
1686	630Δ*erm* Δ*cwlD*-p	630Δ*erm*Δ*cwlD* with *pyrE* restored	This study
1689	630Δ*erm* Δ*pdaA*-p	630Δ*erm*Δ*pdaA* with *pyrE* restored	This study
1692	630Δ*erm* Δ*cwlD*/*cwlD*	630Δ*erm*Δ*cwlD* with *cwlD* in the *pyrE* locus	This study
1695	630Δ*erm* Δ*pdaA*/*pdaA*	630Δ*erm*Δ*pdaA* with *pdaA* in the *pyrE* locus	This study
***E. coli* strains**			
41	DH5α	F^- Φ80*lacZ*ΔM15 Δ(*lacZYA-argF*) U169 *recA1 endA1 hsdR17* (rK^-, mK^+) *phoA supE44* λ– *thi-1 gyrA96 relA1*	D. Cameron
531	HB101/pRK24	F^- *mcrB mrr hsdS20*(rB^-mB^-) *recA13 leuB6 ara-13 proA2 lavYI galK2 xyl-6 mtl-1 rpsL20* carrying pRK24	C. Ellermeier
892	BL21 (DE3)	F^- *ompT hsdSB*(rB^-mB^-) *gal dcm* (DE3)	C. Ellermeier
1338	pET22b-*pdaA*	pET22b-*pdaA* in BL21(DE3)	This study
1375	pET28a-*cspBA*+TAA	pET28a-*cspBA* including its stop codon in DH5α	This study
1539	pMTL-YN3	pMTL-YN3 in DH5α	[1]
1604	pET29a-Δ22-*gerS*	pET29a-*gerS* (deletion of N-terminal 26 aa) in DH5α	[3]
1617	pET22b-empty vector + pET29a-Δ22-*gerS*+TAA	Co-expression of pET22b empty vector with pET29a-*gerS*+TAA (untagged deletion of N-terminal 26 aa) in BL21(DE3)	This study
1660	pMTL-YN3-Δ*sleC*	pMTL-YN3-Δ*sleC* in HB101/pRK24	[2]
1662	pMTL-YN1C	pMTL-YN1C in HB101/pRK24	[1]
1747	pMTL-YN1C-*gerS**	pMTL-YN1C-*gerS* in HB101/pRK24	This study
1933	pMTL-YN1C-*gerS*	pMTL-YN1C-*gerS-alr2* in HB101/pRK24	This study
1941	pMTL-YN3-Δ*cwlD*	pMTL-YN3-Δ*cwlD* in HB101/pRK24	This study
1944	pMTL-YN3-Δ*pdaA*	pMTL-YN3-Δ*pdaA* in HB101/pRK24	This study
1969	pMTL-YN1C-*cwlD*	pMTL-YN1C-*cwlD* in HB101/pRK24	This study
1970	pMTL-YN1C-*pdaA*	pMTL-YN1C-*pdaA* in HB101/pRK24	This study
2045	pET22b-Δ25-*cwlD*-His_6	pET22b-*cwlD* (deletion of N-terminal 25 aa with His_6 tag) in BL21(DE3)	This study
2072	pET22b-Δ25-*cwlD*-His_6 + pET29a-empty vector	Co-expression of pET22b-*cwlD* (deletion of N-terminal 25 aa with His_6 tag) with pET29a-empty vector in BL21(DE3)	This study

2073	pET22b-Δ25-*cwlD*-His_6 + pET29a-Δ22-*gerS* +TAA	Co-expression of pET22b-*cwlD* (deletion of N-terminal 25 aa with His_6 tag) with pET29a-*gerS* (untagged deletion of N-terminal 26 aa) in BL21(DE3)	This study
2074	pET22b-Δ25-*cwlD*-His_6 + pET28a-*cspBA*+TAA	Co-expression of pET22b-*cwlD* (deletion of N-terminal 25 aa with His_6 tag) with pET28a-*cspBA* (untagged) in BL21(DE3)	This study
**Plasmids**		**Relevant features**	
	pET22b	*amp*	Novagen
	pET28a	*kan*	Novagen
	pET29a	*kan*	Novagen
	pK424	Tra^+ Mob^+; *bla*, *tet*	C. Ellermeier

References

1. Ng YK, Ehsaan M, Philip S, Collery MM, Janoir C, Collignon A, et al. Expanding the repertoire of gene tools for precise manipulation of the *Clostridium difficile* genome: allelic exchange using *pyrE* alleles. PLoS One. 2013;8(2):e56051. doi: 10.1371/journal.pone.0056051. PubMed PMID: 23405251; PubMed Central PMCID: PMCPMC3566075.
2. Donnelly ML, Li W, Li YQ, Hinkel L, Setlow P, Shen A. A *Clostridium difficile*-Specific, Gel-Forming Protein Required for Optimal Spore Germination. mBio. 2017;8(1). Epub 2017/01/17. doi: 10.1128/mBio.02085-16. PubMed PMID: 28096487; PubMed Central PMCID: PMCPMC5241399.
3. Fimlaid KA, Jensen O, Donnelly ML, Francis MB, Sorg JA, Shen A. Identification of a Novel Lipoprotein Regulator of *Clostridium difficile* Spore Germination. PLoS Pathog. 2015;11(10):e1005239. doi: 10.1371/journal.ppat.1005239. PubMed PMID: 26496694; PubMed Central PMCID: PMCPMC4619724.
