# Supplementary material for: Clostridium difficile Lipoprotein GerS Is Required for Cortex Modification and Thus Spore Germination
Source: mSphere. 2018 Jun 27;3(3):e00205-18. doi: 10.1128/mSphere.00205-18 (PMC6021603; doi:10.1128/mSphere.00205-18)
Supplement: TABLE S3 [file sph003182575st3.pdf]

Table S3. Primers used in this study.

Primer	Name	Sequence
1278	5' NdeI *gerS* truncation	AGC**CATATG**CAAAAACGACAGTCCAC
1458	5' NdeI *pdaA* start	ATA**CATATG**AAAAAAGATAGCCTAAAAAAATACATTATGATAGG
1459	3' XhoI *pdaA* no stop	AAT**CTCGAG**TTTTTTATTATTTAAATAATCAAGTGTTTTAAGTTTATAACCTTC
1505	5' NcoI *cspB* codon opt	ATA**CCATGG**CTATTATCATTAATTACGAACTGATTGTGAAGTAC
1507	5' linker *cspA* SOE	GACGAGGAAAATAGCTACAAGTTTATCGATGGATACAACATTCAGATTCATAACGATC
1508	3' XhoI *cspA* codon opt no stop	AAT**CTCGAG**GCGCAGTACATCAAACATGCCACG
1529	3' HindIII *cspBA* CO 2439	TTTGAAGCTTTTATTAATGCTGCGATAGCC
1530	5' HindIII *cspBA* linker 2482	TAAAAGCTTCAAAGACGAGGAAAATAGCTACAAGTTTATCGATGGA
1625	3' XhoI *cspBA* codon opt with stop	AAT**CTCGAG**TTAGCGCAGTACATCAAACATGCCACG
1900	3' XhoI *gerS*+TAA	AAT**CTCGAG**TTAGTTTCTGTATTCAAAATCTTTGTATTTTAC
2003	5' *gerS* flank	GCTTAGTTGTGTAGTATAAT
2004	3' *gerS* flank	GTATAACCTCATCTCCA
2005	5' SOE Δ*gerS*	GACCATAGTATGTATTATGTTCTTGGCAGTAAAATACAAAGATTTTGAATACAGAAAC
2006	3' rev oes Δ*gerS*	GTTTCTGTATTCAAAATCTTTGTATTTTACTGCCAAGAACATAATACATACTATGGTC
2007	5' AscI Δ *gerS* Gibson	GTCAATTGTTCAAAAAAATAATGGCG**GCGCGCC**GCTTAGTTGTGTAGTATAATA
2008	3' SbfI Δ *gerS* Gibson	AGCAAGGCAAGACCGATCGGGCCC**CCTGCAGG**CTTTGTCAATTCTAACCA
2181	5' NotI *gerS*	GGAATTAGGGATGTAATAA**GCGGCCGC**CAGTTGTAGATTCAGAGAATAGAGTTG
2182	3' XhoI CD3464 Gibson	GCCAAGCTTGCATGTCTGCAGGC**CTCGAG**TTAGTTTCTGTATTCAAAATCTTTGTATTTT
2362	5' NotI *cwlD* 263	GGAATTAGGGATGTAATAAG**CGGCCGC**ATAAGGTTATATATTTTAAGATATTATTTAAC
2387	5' *cwlD* flank 850	GCTAGAGTAAGACTAGTTGCAGGTG
2388	3' *cwlD* flank 861	GCTAGAGTAAGACTAGTTGCAGGTG
2389	5' AscI Δ*cwlD* YN3	GTCAATTGTTCAAAAAAATGGC**GGCGCGCC**GACATTAATAAGAGATGTTAAACAACC
2390	5' Δ*cwlD* SOE	GCAATGATATGTCTAGTAGTAGTATCCGAGTGTAAACTCTTAACTGATGAAACA
2391	3' Δ*cwlD* rev eos	TGTTTCATCAGTTAAGAGTTTACACTCGGATACTACTACTAGACATATCATTGC
2392	3' SbfI Δ*cwlD* YN3	AGCAAGGCAAGACCGATCGGGCCC**CCTGCAGG**GTCATTATCTTCCTTGAGGACAGAG
2408	3' XhoI *gerS-alr2*	CAAGCTTGCATGTCTGCAGGC**CTCGAG**TTATTTTAGCAAATAACTGTTTATTTG
2428	5' *pdaA* flank 800	GCTCCATTCCCATGTTGGAAG
2429	3' *pdaA* flank 879	CATAGAAGGAGGATATCTGCTTG
2430	5' Ascl Δ*pdaA* YN3 Gibson	GTCAATTGTTCAAAAAAATAATGGC**GGCGCGCC**CTCAATGGCTCCAAAGTAGTTTTTC
2431	5' Δ*pdaA* SOE	GCTCTAATACTTTTTGGAATTGCTAGGATGAGGTTCTTACACATTGGG
2432	3' Δ*pdaA* rev eos	CCCAATGTGTAAGAACCTCATCCTAGCAATTCCAAAAAGTATTAGAGC
2433	3' Sbfl Δ*pdaA* YN3 Gibson	GCAAGGCAAGACCGATCGGGCCC**CCTGCAGG**GAATTTTGGCAATATGTATCACATGAAG
2450	3' XhoI *cwlD* YN1C	CAAGCTTGCATGTCTGCAGGC**CTCGAG**TCAACTTAAATATTTTTGTATTCCTATGTAG
2451	5' NotI *pdaA* YN1C	CAAGGAATTAGGGATGTAATAA**GCGGCCGC**CCAGCCTAACTTATTTACAATCATTTCTC
2455	5' Δ*cwlD* internal colony PCR	GAGCTTATAGAATCAAGTGGTGGCC
2457	3' XhoI *pdaA* YN1C	CAAGCTTGCATGTCTGCAGGC**CTCGAG**GGATACAAAGAGATAAATGAATGGAC
2498	5' Δ*pdaA* internal colony PCR	CATCCATCAATGGCAGGTATTAC
2545	5' *cwlD*$_{\Delta 25aa}$ His$_6$ pET22b	GTTTAACTTTAAGAAGGAGATATA**CATATG**AAAAATATTTCTGAAGATGTTATCAAG
2546	3' *cwlD*$_{\Delta 25aa}$ His$_6$ pET22b	GCTCGAGTGCGGCCGCAAGCTT**GTCGAC**ACTTAAATATTTTTGTATTCCTATGTAGATTG

Restriction sites are underlined and in bold.
